# A Clinical Update on SARS-CoV-2: Pathology and Development of Potential Inhibitors

**DOI:** 10.3390/cimb45010028

**Published:** 2023-01-04

**Authors:** Desh Deepak Singh, Ihn Han, Eun-Ha Choi, Dharmendra Kumar Yadav

**Affiliations:** 1 Amity Institute of Biotechnology, Amity University Rajasthan, Jaipur 303002, India; 2 Plasma Bioscience Research Center, Applied Plasma Medicine Center, Department of Electrical & Biological Physics, Kwangwoon University, Seoul 01897, Republic of Korea; 3 Department of R&D Center, Arontier Co., Seoul 06735, Republic of Korea

**Keywords:** SARS-CoV-2, variant, antibody, treatment, efficacy, neutralization therapy, drug treatment

## Abstract

SARS-CoV-2 (severe acute respiratory syndrome) is highly infectious and causes severe acute respiratory distress syndrome (SARD), immune suppression, and multi-organ failure. For SARS-CoV-2, only supportive treatment options are available, such as oxygen supportive therapy, ventilator support, antibiotics for secondary infections, mineral and fluid treatment, and a significant subset of repurposed effective drugs. Viral targeted inhibitors are the most suitable molecules, such as ACE2 (angiotensin-converting enzyme-2) and RBD (receptor-binding domain) protein-based inhibitors, inhibitors of host proteases, inhibitors of viral proteases 3CLpro (3C-like proteinase) and PLpro (papain-like protease), inhibitors of replicative enzymes, inhibitors of viral attachment of SARS-CoV-2 to the ACE2 receptor and TMPRSS2 (transmembrane serine proteinase 2), inhibitors of HR1 (Heptad Repeat 1)–HR2 (Heptad Repeat 2) interaction at the S2 protein of the coronavirus, etc. Targeting the cathepsin L proteinase, peptide analogues, monoclonal antibodies, and protein chimaeras as RBD inhibitors interferes with the spike protein’s ability to fuse to the membrane. Targeting the cathepsin L proteinase, peptide analogues, monoclonal antibodies, and protein chimaeras as RBD inhibitors interferes with the spike protein’s ability to fuse to the membrane. Even with the tremendous progress made, creating effective drugs remains difficult. To develop COVID-19 treatment alternatives, clinical studies are examining a variety of therapy categories, including antibodies, antivirals, cell-based therapy, repurposed diagnostic medicines, and more. In this article, we discuss recent clinical updates on SARS-CoV-2 infection, clinical characteristics, diagnosis, immunopathology, the new emergence of variant, SARS-CoV-2, various approaches to drug development and treatment options. The development of therapies has been complicated by the global occurrence of many SARS-CoV-2 mutations. Discussion of this manuscript will provide new insight into drug pathophysiology and drug development.

## 1. Introduction

From January 2020 until now, the development of SARS-CoV-2 has accelerated, and hundreds of candidate medications have been tested in preclinical and clinical stages for use against the virus [1,2,3]. The clinical trials that examine novel COVID (coronavirus disease) therapies provide crucial information about their efficacy and safety [3,4]. The FDA (Food and Drug Administration), EMA (European Medicines Agency), or other national regulatory bodies must approve clinical trials [5]. Several medicines are currently undergoing phase 3 and 4 clinical trials [4,5]. Antiviral agents, antibodies, cellular molecules, RNA-based drugs, and repurposing therapies have all been mentioned as preclinical therapeutic candidates [6]. Other methods of treatment include antimalarial, proteinaceous, interferon, anti-inflammatory, and antibiotic receptor modifying drugs [7]. As of 30 September 2022, there were more than 700 drug development programmes in various stages of preparation, and the FDA had evaluated more than 450 trials [8]. Since the outbreak of SARS-CoV-2 in early 2020, experts have been working to develop medicines that can cure the SARS-CoV-2 infection [8,9]. SARS-CoV-2 variants are spreading all over the world, with some of them playing a key role in increasing contagiousness, toxicity, lowering the effectiveness of SARS-CoV-2 treatment choices, and leading to the declaration of the COVID-19 pandemic [10]. Increased infectivity of SARS-CoV-2 is associated with higher spike protein–human ACE2 receptor-binding affinity. According to research on structures and functions [11], the SARS-CoV-2 S protein binds to ACE2 10-fold more potently than the SARS-CoV S protein. The mutation N501Y was discovered in B.1.1.7 (alpha variant), which improved ACE binding affinity. The binding effectiveness of ACE2 with N501Y, K417N, and E484K was altered by B.1.351 (beta variant) [10,11]. It can be challenging to find and create SARS-CoV-2 medications that target the spike protein molecule, TMPRSS2 protease, or the ACE2 receptor, for example, because of its volatile chemical compounds [10,11]. Option B.1.1.529 was first reported to the World Health Organization (WHO) on 24 November 2021, in South Africa [11]. Each of these variations have mutations that increase viral toxicity and immunosuppression. Because binding of SRBD (receptor-binding-domain of spike) to host cell ACE2 receptors is the initial stage in the SARS-CoV-2 virus’s life cycle, effective medicines against COVID-19 should predominantly target viral SRBD [8,11]. SARS-RBD CoV-2’s domain is a competitive inhibitor of the ACE2 receptor-binding site. The US Food and Drug Administration (FDA) approves drug efficacy through drug design, clinical trials, and emergency use [10,11]. The SRBD crystal structure containing the Wuhan Hu1 sequence is effective for drug development for SARS-CoV-2. In the SARS-CoV-2 pandemic, a variety of pharmacological techniques were used, including network models, structural approaches, and machine/deep learning approaches [12]. The drug reproduction system is designed in contrast to typical medication development, such as compound recognition, compound purchase, FDA pre-design and approval, and product safety and distribution [8,13]. In the early stages of SARS-CoV-2 pathogenesis, the focus was on the SARS-CoV-2 CLpro enzyme, host protease, inhibition of SARS-CoV-2 virus adhesion to the ACE2 receptor, HR1–HR2 association with the coronavirus S2 (spike 2) protein, and cathepsin L protease target. Early research focused on TMPRSS2-related inhibitors, membranes, peptide analogues, monoclonal antibodies, and protein chimaeras as RBD inhibitors. Then, using a variety of approaches, various antiviral medications were comprehensively screened, allowing RNA-dependent RNA polymerase (RdRp) to emerge as an intriguing target for the development of antiviral treatments against SARS-CoV-2 [14,15,16]. SARS-CoV-2 as RdRp has produced the first verified target for the development of novel antiviral medications [14,15,16], thereby addressing current medical demands [13]. In addition, even though several clinical trials are currently underway, the evaluation of the potential efficacy of various active components that have been shown to have complete drug reuse is still incomplete [15,16]. A small number of mutations have also appeared multiple times in the overall mutation population, indicating that certain mutations have significant adaptive advantages [17]. Here, we discuss the clinical update on pathophysiology and the development of potential inhibitors for SARS-CoV-2.

## 2. Pathology

### 2.1. Clinical Characteristics and Diagnosis of SARS-CoV-2 Infection

**Figure 1:**
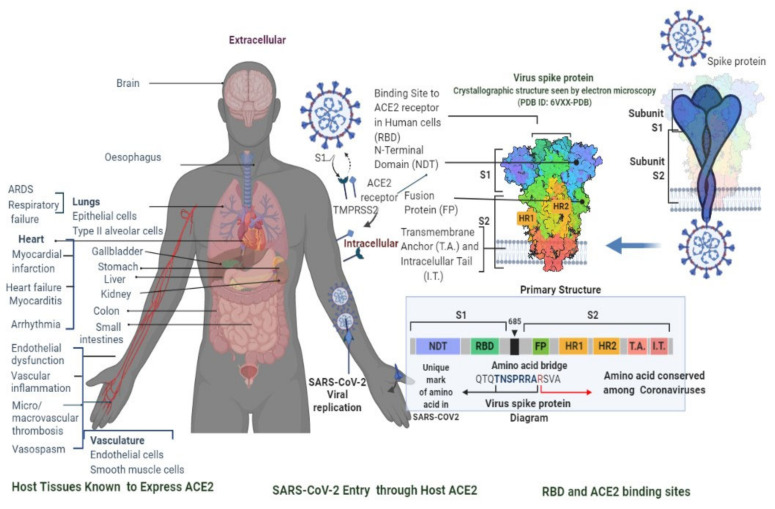
When an infected person releases the virus, it connects to ACE2 receptors on airway host cells and spreads. Targeting and invasion of ACE2 receptor-infected cells by SARS-CoV-2, as well as ACE2 receptor expression in human host tissues. SARS-CoV-2 S glycoprotein is made up of two subunits, S1 and S2, which are commonly depicted as a sword-like tip. The glycoprotein model from the Protein Data Bank (PDB) shows how subunits are arranged in several critical areas for the infectious process. An amino acid bridge connects proteins S1 and S2. This is vital information when considering virus targeting. Since it is essential for viral attachment, fusion, and invasion, the CoV-Spike (S) protein is a target for the creation of inhibitors. In both humans and bats, there is a close relationship between the RBD (receptor-binding domain) protein and the ACE2 receptor. In contrast to SARS-CoV RBD, SARS-CoV-2 RBD has a considerably higher affinity for binding to ACE2 receptors, which prevents either virus from attaching to or connecting to ACE2-expressing cells and transmitting infection to host cells. Randomisation of trials and at least one risk factor (heart disease, diabetes, etc.) are linked to worse illness outcomes.

**Figure 2:**
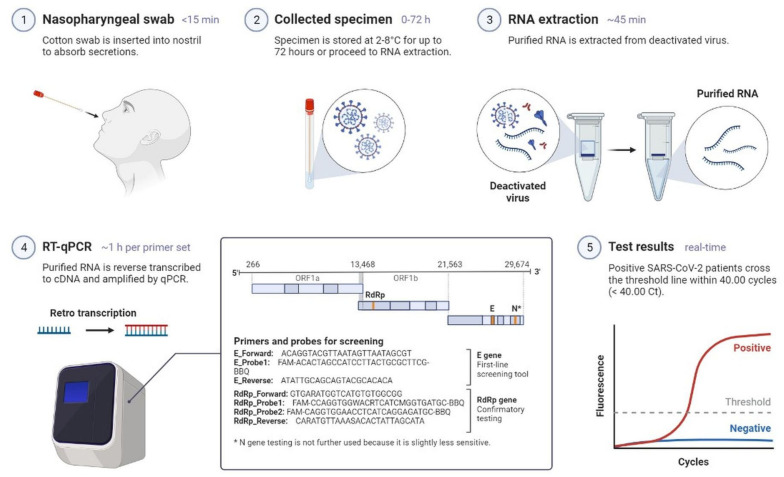
Representation of COVID-19 diagnostic test through RT-PCR.

**Figure 3:**
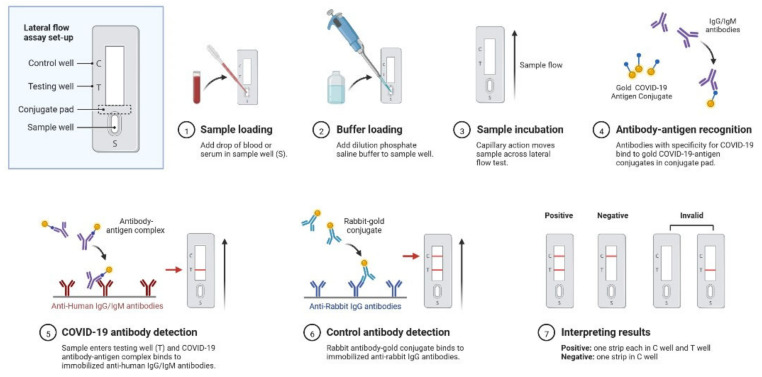
Representations of COVID-19 serologic diagnostic testing through antibody detection. It depicts sample loading, SARS-CoV-2 antibody-antigen detection, and qualitative test results.

**Figure 4:**
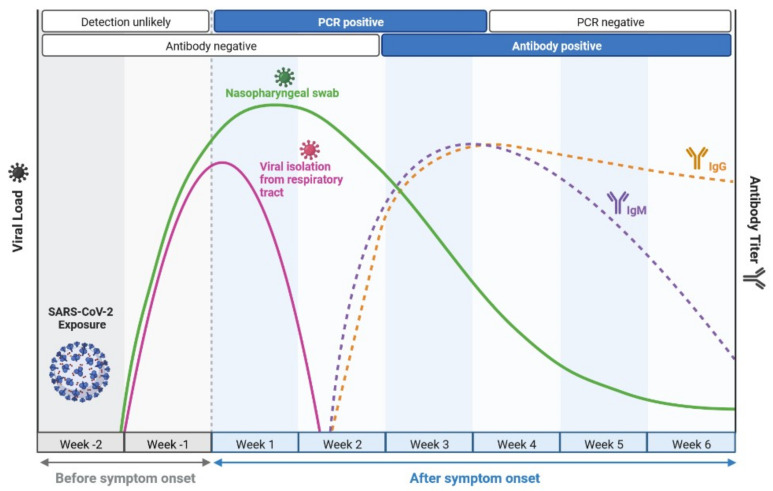
A time course of COVID infection and test positivity by either PCR-based or serological (antibody titre) testing. Notably, PCR tests are positive earlier in disease course, and serological tests are positive later in the disease course.

SARS-CoV-2 causes an instantaneous viral infection in humans with an incubation period of 3–10 days [18]. A comparison of the epidemiological, clinical, and radiological characteristics of SARS-CoV, MERS-CoV, and SARS-CoV-2 diseases is shown in Table 1 [18,19,20]. COVID-19 causes adults to show obvious symptoms such as cough (67.7%), fever (87.9%), fatigue (38.1%), vomiting (5.0%), and diarrhoea (3.7%); these are also seen in other coronavirus disorders [19,20]. In the original instances of infection, there was only a 9-day lag between the onset of symptoms and the diagnosis of acute respiratory distress syndrome [18,19], and most patients had dyspnoea. Complications are frequently a risk for patients who have experienced significant events, such as acute respiratory failure, severe heart failure, or a subsequent infection (Figure 1) [20]. Furthermore, COVID-19 also damages organs and tissues apart from the lungs (Figure 1) [18,19,20]. SARS-CoV-2 illness is frequently misdiagnosed as influenza or a seasonal upper respiratory tract virus infection [18,20]. Early diagnosis is necessary for the clinical management of COVID-19 cases. The rapid and precise identification of SARS-CoV-2 made possible by a real-time reverse transcription-polymerase chain reaction (RT–PCR) is the first step towards managing COVID-19 from nasopharyngeal fluid (Figure 2) [21]. Serological testing complements virus detection as it indicates past infection, which could be harnessed for therapeutic gain. Antibodies are detected by an enzyme-linked immunosorbent assay using a qualitative detection of IgG or IgM antibodies (Figure 3) [21]. RT-PCR tests are sensitive to SARS-CoV-2 infection, but they sometimes miss cases. Serological tests are positive later in the disease course, after PCR tests show negative results (Figure 4) [20,21]. Radiological testing is also recommended after the onset of respiratory problems and for nucleic acid viral detection techniques. These tests could involve a lung ultrasound, a CT scan, or a chest X-ray [20,21]. These can also be employed at different stages of the SARS-CoV-2 infection, either individually or simultaneously.

### 2.2. Immunopathology

**Figure 5:**
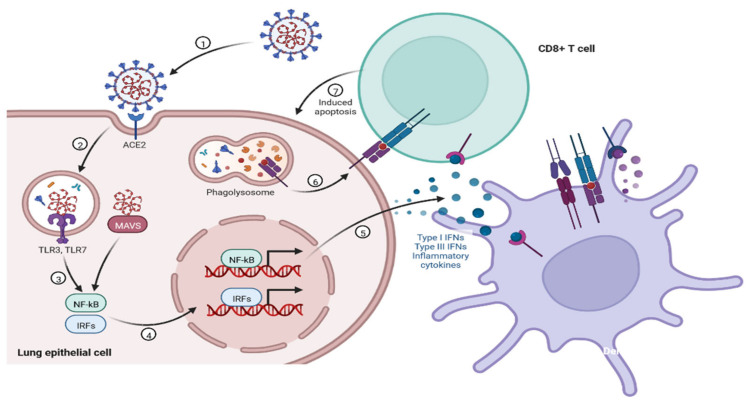
Coronaviruses are RNA viruses, some of which can infect human lung epithelium via the receptor ACE2. Viral RNA activates the endosomal and cytoplasmic sensors, TLR3/7 and MAVS, respectively. These receptors activate Interferon Regulatory Factors (IRFs) and NFkB to induce inflammatory cytokines, including interferons (IFN). Dendritic cells (DCs) sample antigen and migrate to lymphoid organs to prime adaptive immunity. CD8 T cells induce apoptosis after recognition of antigen on DCs or infected cells.

**Figure 6:**
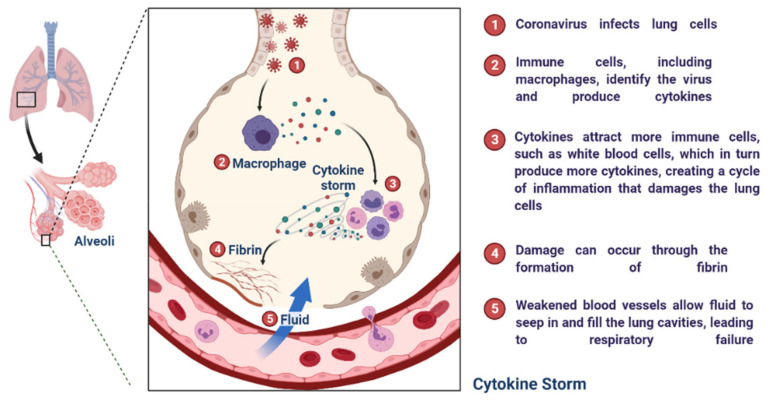
Cytokine storm: When cytokines are triggered without breaks, they can cause damage to the cells responding to the cytokines and shut down the function of the organs. This is known as a cytokine storm, which mediates severe disease, including COVID-19.

**Figure 7:**
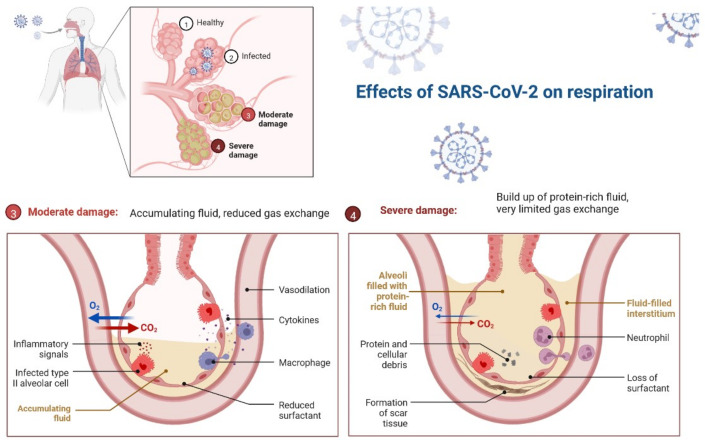
After entering the body through the mouth or nose, SARS-CoV-2 makes its way into the lungs, where it uses its distinctive spike proteins to infect alveolar cells. In response, the immune system attacks the area of infection, killing healthy alveolar cells in the process. Reduced surfactant from alveolar epithelial type II cells, along with increased fluid accumulation in the alveoli, causes reduced or severely hindered gas exchange.

**Figure 8:**
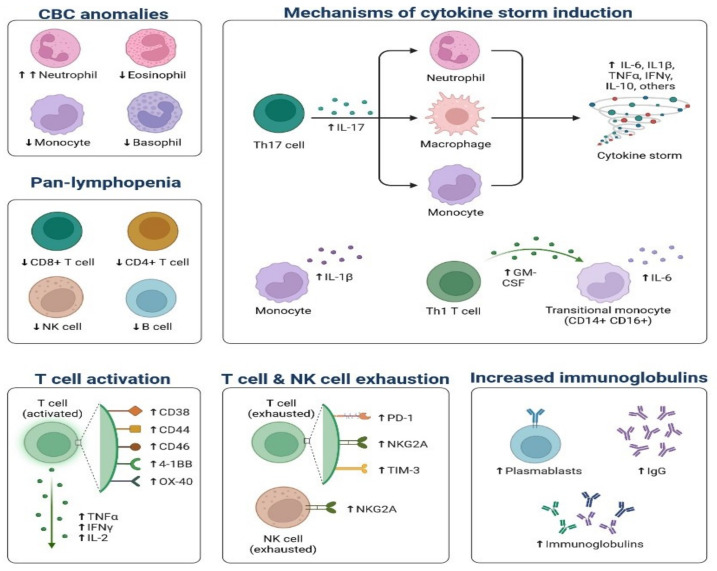
Recruitment of T and B Cells by APCs: Once they reach the target tissue, killer T cells detect specific virus fragments on the surface of infected cells and destroy them, eliminating virus factory. Antibodies secreted by B cells bind to the surface of the virus and block host entry (neutralizing antibodies).

**Figure 9:**
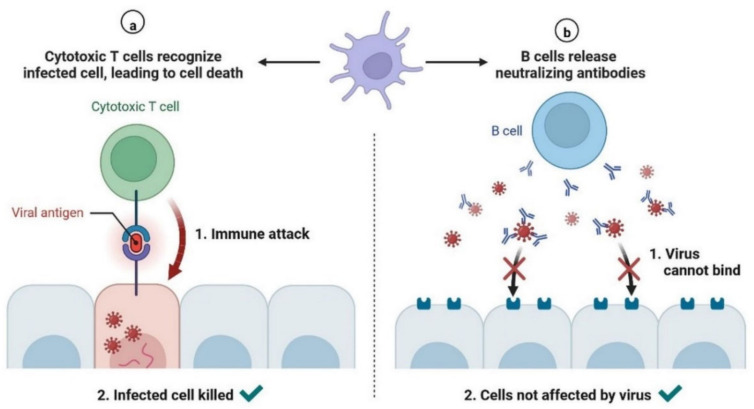
Immune Mechanisms Affected by COVID-19: (**a**) Cytotoxic T cells recognise infected cells, leading to cell death. (**b**) B cells release neutralising antibodies, which prevent the binding of the virus.

During SARS-CoV-2 infection, the virus infects and replicates in the lung epithelial cells [23]. Coronaviruses infect human lung epithelium via the receptor ACE2 and activate the endosomal and cytoplasmic sensors, TLR3/7 and MAVS, respectively. These receptors trigger the expression of interferon regulatory factors (IRFs) and NFkB (Figure 5). The virus is detected by microphases through pattern recognition receptors, which trigger the secretion of interferons and cytokines (Figure 6) [24]. However, the virus appears to be immune to the antiviral effects of interferon; instead, the cytokines cause more WBCs to swarm the tissue, resulting in a cytokine storm in which many cells contribute to the secretion of a variety of cytokines (Figure 6 and Figure 7) [25,26]. Some of these cytokines include fibrin deposits as well as damage to the blood vessels, causing fluid to leak into the alveoli, which causes respiratory failure [24,25,26]. When viruses block the secretion and function of cytokines and interferons, allowing them to replicate and avoid destruction by invading the host’s innate defence system, the body initiates the second layer of the immune system, i.e., the adaptive immune response [26]. The T and B lymphocytes are the key players in the adaptive immune system, but SARS-CoV-2 infection reduces the T cells, monocyte, natural killer, and dendritic cells (DCs) (Figure 8 and Figure 9) [27]. Once dendritic cells detect viruses through the pattern recognition receptors, they not only secrete cytokines but are also signalled to migrate to draining lymph nodes [26]. Naive, unnucleated T and B cells located in the lymph nodes interact with dendritic cells. If their receptors happen to match the antigen presented by the dendritic cells, they are activated, expand, and become so-called effector lymphocytes (Figure 8) [25,26,27]. These effector lymphocytes migrate back to the site of infection and directly kill infected cells and block further viral spread by secreting specific antibodies [28]. These specialized WBCs, T cells, and B cells are activated by antigen-presenting cells. Once they reach the target tissue, T cells trained as killer soldiers detect specific virus fragments on the surface of infected cells and destroy them, eliminating the source of viral production [29]. The antigen-presenting cells (APCs) play an important role in the development of SARS-CoV-2 disease [25]. SARS-CoV-2 binds with the target cells and activates the granulocytes, macrophages, dendritic cells, and other immune cells, releasing a complex array of pro-inflammatory cytokines and activating the cellular immune response [26]. B cell activation and antibody hypersecretion cause immune system overreaction, which results in tissue destruction. T cells lead to the development of neutrophils and monocytes in infection, causing lung tissue damage and clinical symptom exacerbation [27,28]. The cytotoxic T cells recognise the infected cell, leading to cell death. B cells produce neutralising antibodies that prevent viruses from attaching to cells (Figure 9) [29]. Virus-binding antibodies secreted by B cells bind to the surface of viruses and block their entry into host cells. These cells are called neutralising antibodies [30]. B cells also secrete non-neutralizing antibodies such as NK cells (natural killer cells) [30,31]. In the adaptive immune system, T and B cells become memory cells and provide immune defence for a long time [31].

## 3. New Variant Detection and Assessment

As shown in Table 2, a few new variants include increased transmissibility, severe disease, or a reduction in neutralisation by antibodies from infection. These variants may also pose a higher risk of eluding testing [32,33]. A variant of high consequence has a demonstrable ability to evade prevention measures or medical countermeasures [34]. Such a variant could potentially elude vaccines or medical treatments. Global field research revealed the emergence of a SARS-CoV-2 variant [35]. More than half of all genomic sequencing of COVID-19 was carried out in the UK. Variants of concern (VOC), variants of interest (VOI), or variants under monitoring (VUM) are shown in Table 3, Table 4 and Table 5 respectively [36,37,38].

## 4. SARS-CoV-2 Drug and Treatment

Therapies for COVID-19 that are being developed include antiviral medications, immunomodulators, treatments using neutralising antibodies, cell therapies, and treatments based on other approaches (Table 6) [39,40]. Our knowledge of the effects of many types of possible treatments is being rapidly investigated [39]. Antiviral drugs are used to treat a variety of viral illnesses and stop viral replication (such as HIV, herpes, hepatitis C, and influenza) [39]. Antiviral Paxlovid (nirmatrelvir and ritonavir) and Lagevrio (molnupiravir) drugs are prescription therapies that fight viruses in the body (used in different forms, including pills, liquid, powder for inhalation, or intravenous solution) [40,41]. Veklury (Remdesivir) is approved for the treatment of COVID-19 in adults and paediatric patients who have positive direct SARS-CoV-2 viral test results, are hospitalized, or are not admitted to a hospital, have mild-to-moderate COVID-19, and are at high risk for progression to severe COVID-19 [41]. Convalescent plasma and specific antibodies, which contain antibodies derived from individuals who have already had COVID-19, as well as antibodies derived from animals and synthetic antibodies, may help patients fight the virus [42]. Cell treatment products include cellular immunotherapies and a variety of autologous and allogeneic cell types [43]. Monoclonal antibodies (mAbs) that specifically target SARS-CoV-2 can support the immune system’s attack on the virus [44,45]. These mAbs prevent the virus from entering human cells and therefore neutralise it [45]. The use of the mAbs Bebtelovimab and Evusheld (tixagevimab co-packaged with cilgavimab) that target SARS-CoV-2 is permitted by an EUA [44]. SARS-CoV-2 can mutate over time, like other contagious organisms, leading to genetic heterogeneity in the population of circulating viral strains [45,46]. Certain variations have the potential to result in resistance to one or more of the mAb treatments approved to treat COVID-19 [46]. The immune system may become overactive in the event of COVID-19 infection, which could cause the disease to worsen. Immune regulators can aid in reducing this inflammation [47]. Olumiant (baricitinib) is approved for the treatment of COVID-19 in children from 2 to under 18 years of age who require extracorporeal membrane oxygenation, invasive mechanical ventilation, or supplemental oxygen (ECMO) [48]. Gene therapy products aim to change a gene’s expression or the biological characteristics of living cells for therapeutic purposes [47]. Treatment options have been approved for emergency use, and phase III and IV clinical trials have been conducted to assess whether an existing approved drug for treating SARS-CoV-2 infection can be used to treat 100 to 1000 people [47,48]. Several pharmacological options are being studied as supportive therapy in patients with SARS-CoV-2 (Table 6) [48]. Here, we will highlight the most frequently discussed medications and therapies for SARS-CoV-2 patients.

### 4.1. Antivirals

#### 4.1.1. Molnupiravir (MK-4482)

**Figure 10:**
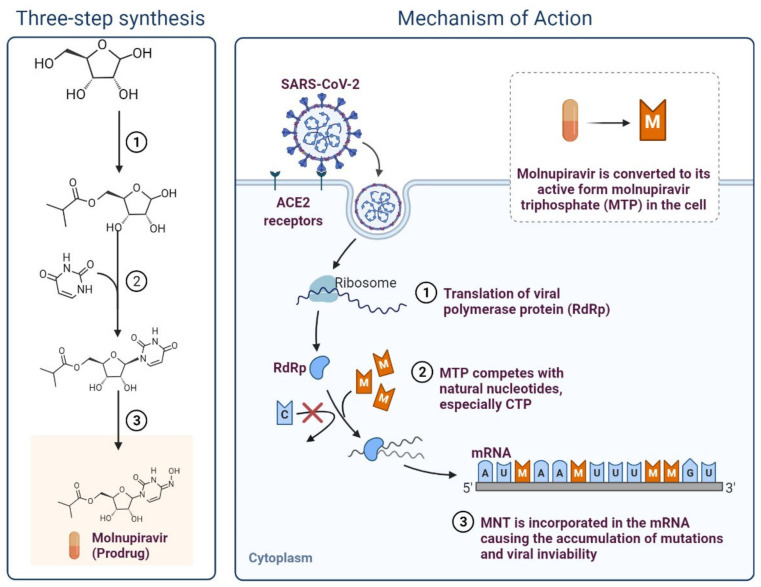
Molnupiravir; Potential repurposed drug candidate for COVID-19.

Molnupiravir (MK4482, EIDD2801) is an oral test version of an effective ribonucleoside analogue that prevents SARS-CoV-2 replication (Figure 10) [62,63]. Molnupiravir has shown effective results in clinical investigations against COVID-19. Merck and Ridgeback began two Phase 2/3 trials in October 2020 to see if this could reduce patient mortality and speed recovery [63]. Promising results were found in hospitalised patients in October 2021 [62]. The approval is based on the positive results of the Phase III clinical study MOVeOUT’s scheduled interim analysis [64]. Uninfected adult patients who were not hospitalised within 5 days of laboratory-confirmed mild to moderate SARS-CoV-2 infection were found to have symptoms. Molnupiravir was investigated in MOVeAHEAD, a global, multicentre, randomised, double-blind, placebo-controlled phase III study, to determine if it could prevent SARS-CoV-2 from spreading within a household. Additionally, post-exposure prophylaxis effects were investigated [64,65]. Following the drug’s post-acquisition market approval under Expanded Access, the FDA issued a Letter of Authority on 11 February 2022. The FDA hosted an ancillary event on 23 March 2022, offering healthcare providers an update to the post-authorization requirements in the Letter of Authorization. Based on the data and perspectives collected to date, the FDA has issued a new Letter of Authorization on 5 August 2022 [64,65].

#### 4.1.2. Favipiravir (Also Known as Avigan)

**Figure 11:**
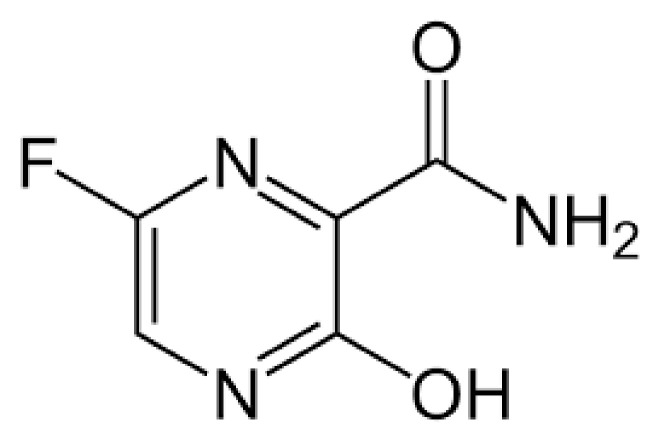
Structure of Favipiravir.

Favipiraviris is a pyrazinecarboxamide derivative that is effective against RNA viruses, as shown in Figure 11. A riboflanosyl triphosphate derivative of favipiravir is produced by a host enzyme and inhibits influenza virus RNA-dependent RNA polymerase [45]. The use of favipiravir in the treatment of SARS-CoV-2 has been reported in some studies, but more evidence is needed to support these findings [65]. Few studies have been carried out on small sample sizes because of the lack of pharmacokinetic and safety data for high-dose favipiravir, which has been suggested as a therapy for SARS-CoV-2. The success of favipiravir has benefited numerous countries and pharmaceutical companies, and approval has been given for SARS-CoV-2 [66]. In patients with severe symptoms, a review of favipiravir research published in February found that it had no effect on death [65]. Research into whether favipiravir can be used as an early treatment for those with SARS-CoV-2 is also ongoing [65]. The efficacy of favipiravir against SARS-CoV-2 mutations needs to be tested in clinical trials [65].

#### 4.1.3. Ritonavir and Darunavir

**Figure 12:**
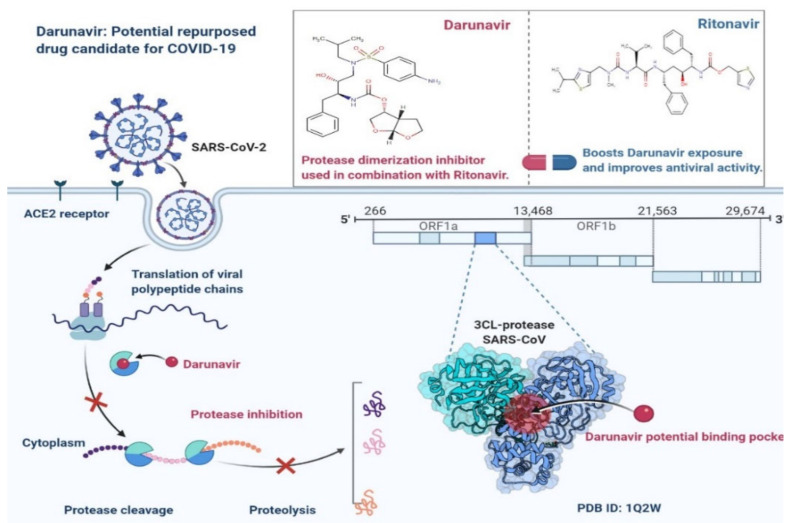
Darunavir: Potential Repurposed Drug Candidate for COVID-19.

Ritonavir and Darunavir (Figure 12) are protease inhibitors used against SARS-CoV-2. They inhibit the enzyme activity required to prevent viral entry into cells [53,66]. Ritonavir is an HIV type 1 aspartate protease inhibitor and is used against SARS-CoV-2 [67]. Ritonavir and Darunavir are combined to increase the plasma half-life. Both drugs are used against SARS-CoV 3C-like protease (3CLpro), which is implicated in the proteolytic mechanism of the replicase polyprotein and is important for viral multiplication [67]. During the SARS-CoV pandemic, the Italian Drug Agency recommended that the drug be used therapeutically based on an effective conclusion from a randomised clinical trial [67]. This HIV (human immunodeficiency virus) drug cocktail was approved by the FDA two decades ago; it also inhibits coronavirus replication in cell cultures (Figure 12) [54]. The WHO stopped research on patients admitted to COVID-19 at the beginning of July 2021. Lopinavir and ritonavir are recommended for both inpatient and outpatient use by the NIH Covid Treatment Guidelines [56]. Lopinavir and ritonavir efficacy against the SARS-CoV-2 mutation has not been demonstrated [66,67]. Darunavir, an unsubstituted amino group at the 4-position N, N-substituted benzene sulphonamide, is used to treat and prevent HIV infection (Figure 12). The lack of specialised antiviral medications for COVID-19 treatment with DRV and SARS-CoV-2 treatment are also obstacles [67].

#### 4.1.4. PAXLOVID™ (PF-07321332)

**Figure 13:**
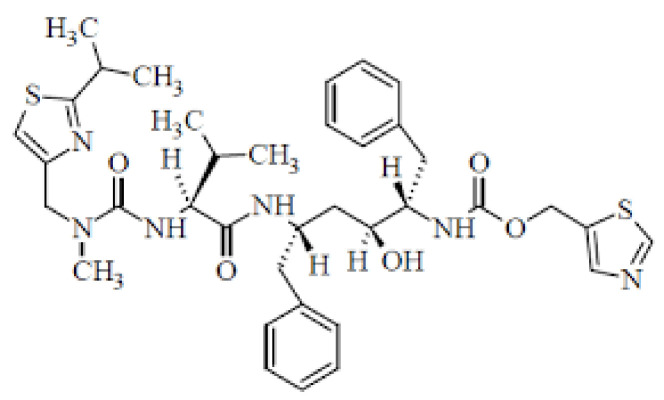
Structure of PAXLOVID™.

Pfizer has developed PF07321332 as a potential treatment for SARS as shown in Figure 13 [68]. At the start of the SARS-CoV-2 pandemic, they modified the drug for intravenous injection; it was originally in pill form. When this drug was administered orally to rats, it reached levels high enough to reduce the coronavirus infection in the patients [68,69]. The clinical trial of PF07321332 was scheduled in March 2021 and a large-scale phase III trial was scheduled in July 2021 [68]. Preliminary results showed an 89% reduction in the probability of hospitalisation or mortality from all-cause COVID-19 compared to placebo in patients treated within 3 days of symptom onset (primary endpoint). At least 0.8% of PAXLOVID^TM^ participants were hospitalised (3/389 were admitted with no deaths). In contrast, 7.0% of patients who received placebos were hospitalised or died (7 follow-up deaths in 27/385) and were hospitalised [68,69]. The results were statistically significant (*p* < 0.0001). In patients treated within 5 days of symptom onset, SARS-CoV-2 showed a comparable trend for hospitalisation or mortality. Patients who received PAXLOVID^TM^ were hospitalised 1.0% of the time prior to 28 days after randomization (6 of 607 patients were hospitalized; no deaths), whereas patients who received placebo were hospitalised 6.7% of the time (41 of 612 patients were hospitalized; 10 subsequent deaths) [68,69]. Prior to 28 days of treatment with PAXLOVID^TM^, there were no reported deaths in the general study population. In contrast, 10 (1.6%) deaths were reported in patients receiving placebo. The recommendation was made in consultation with the FDA and the independent data monitoring committee. Earlier, a total of 1219 adults who had been enrolled up until 29 September 2021 were the subjects of a primary study of the data set [69]. Enrolled participants must have suffered mild to severe symptoms for at least five days after being diagnosed with SARS-CoV-2 and must have had at least one trait or underlying medical condition that raised the risk of them developing serious COVID-19. For a period of five days, every patient was given either PAXLOVID^TM^ or a placebo orally. A large cohort of 1881 EPIC-HR individuals who had data available at the time of the investigation was used to evaluate safety information [68,69]. Contrary treatments were equal in intensity for PAXLOVID^TM^ (19%) and placebo (21%), with most of them being mild. Fewer patients who were examined for treatment of adverse events considered study medication use (2.1% vs. 6.6%) and adverse events (1.7% vs. 6.6%), and 4.1% in PAXLOVID^TM^-treated patients compared to placebo-treated patients. Pfizer’s Equality of Access Commitment is dedicated to ensuring that all patients have equal access to PAXLOVID^TM^ by working to make safe and effective antiviral medications available swiftly and at a reasonable cost [68,69]. It has shown substantial antiviral action in vitro against circulating strains of concern as well as other known coronaviruses, implying that it could be used to treat a variety of coronavirus infections. While waiting for favourable trial findings and regulatory approval, the company is trying to guarantee that those in need around the world have access to novel antiviral medications [69]. The replication of SARS-CoV-2 is inhibited by CYP3A-mediated metabolism. After investigation of promising results, the FDA has issued new Letters of Authorization (LOA) on 17 March 2022, 14 April 2022, and 6 July 2022 [69]. On 5 August 2022, the FDA has issued a new authorization letter based on clinical trial results in immunocompromised patients with mild-to-moderate disease.

#### 4.1.5. Ivermectin

**Figure 14:**
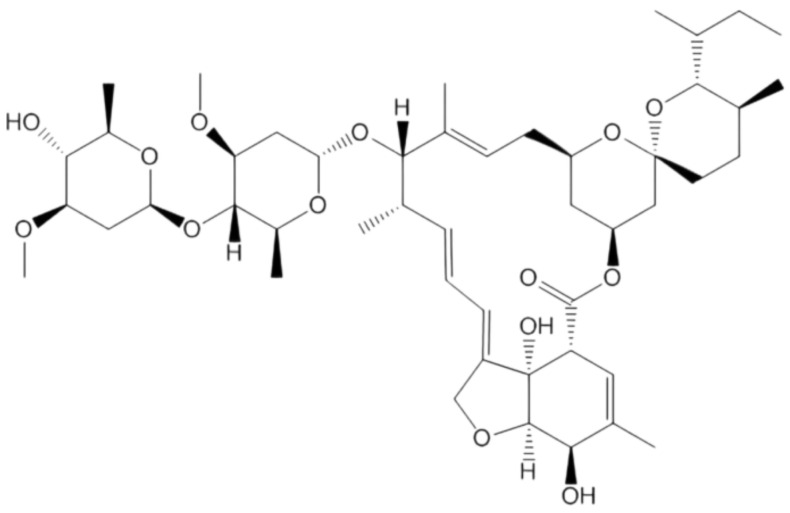
Structure of Ivermectin.

Ivermectin has been an effective drug for the treatment of parasites as shown in Figure 14. Researchers in Australia reported that the drug blocked the coronavirus in cell cultures but used doses high enough to have dangerous side effects in humans, causing an immediate warning against the use of veterinary drugs containing ivermectin by the FDA [70,71,72]. Ivermectin was further evaluated in July 2021 in a large-scale, randomized clinical trial and provided more accurate data [72,73]. However, later clinical research showed that Ivermectin is not a potential inhibitor of SARS-CoV-2 and Kumar et.al demonstrated a cell line [71]. The National Institutes of Health has begun testing for medicines in patients over 30 years of age who have tested positive for SARS-CoV-2 in the previous 10 days and have had at least two symptoms in the previous week [70,71]. Immediately before the start of this study, another study of 1500 patients showed no effect of ivermectin. Ivermectin has five different mechanisms of action on coronavirus, and the drug is also effective against various variants of the virus. The dose of ivermectin was adjusted with the protocol and additional drugs and measures were added to make the protocol more effective for the variant [72].

#### 4.1.6. Remdesivir

**Figure 15:**
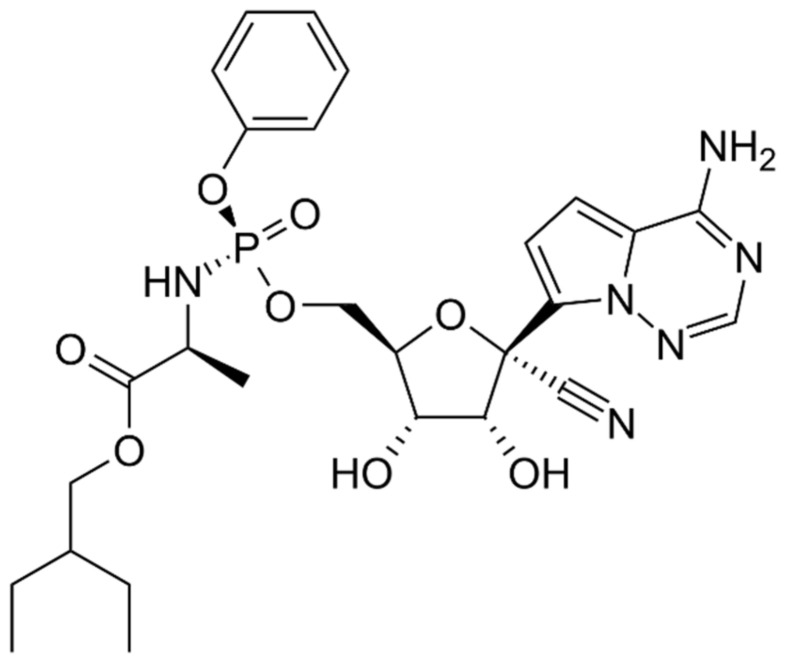
Structure of Remdesivir.

Remdesivir is used as an antiviral agent against hepatitis C virus and Ebola virus as shown in Figure 15 [49]. Clinical trials were conducted on SARS-CoV-2 to reduce recovery time for in patients. The FDA was granted full drug approval on 8 October 2020 [49,50,51]. This licence does not apply to the full population that was permitted to use Veklury under an EUA that was initially issued on 1 May 2020. Veklury’s use for the treatment of suspected or laboratory-confirmed COVID-19 in hospitalised paediatric patients weighing from 3.5 kg to less than 40 kg, or hospitalised paediatric patients younger than 12 years of age weighing at least 3.5 kg, is now authorised by the FDA after a revised EUA for the medication [73]. This was completed to maintain access to the paediatric population that the EUA had previously covered. Clinical trials investigating Veklury’s effectiveness and safety in this patient population are currently being conducted [74]. The World Health Organization raised more scepticism when it published a global randomised study that found that remdesivir had little or no effect on hospital stay, ventilation risk, or mortality from all causes. Based on these results, WHO has issued guidelines recommending the use of remdesivir [74]. The National Institutes of Health recommends using remdesivir only in patients who are hospitalised and need oxygen. They advise seriously ill patients not to take the drug [74].

#### 4.1.7. Oleandrin

**Figure 16:**
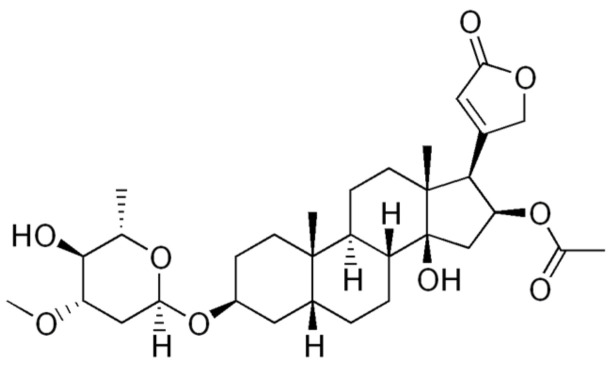
Structure of Oleandrin.

The oleander bush produces a compound called oleandrin, as shown in Figure 16. Researchers from Phoenix Biotechnology, a San Antonio-based company, and Galveston University of Texas published a study demonstrating that coronavirus-infected monkey kidney cells are protected from SARS-CoV-2 [75]. Oleandrin’s effectiveness against SARS-CoV-2 mutants has not been studied.

#### 4.1.8. Dexamethasone

**Figure 17:**
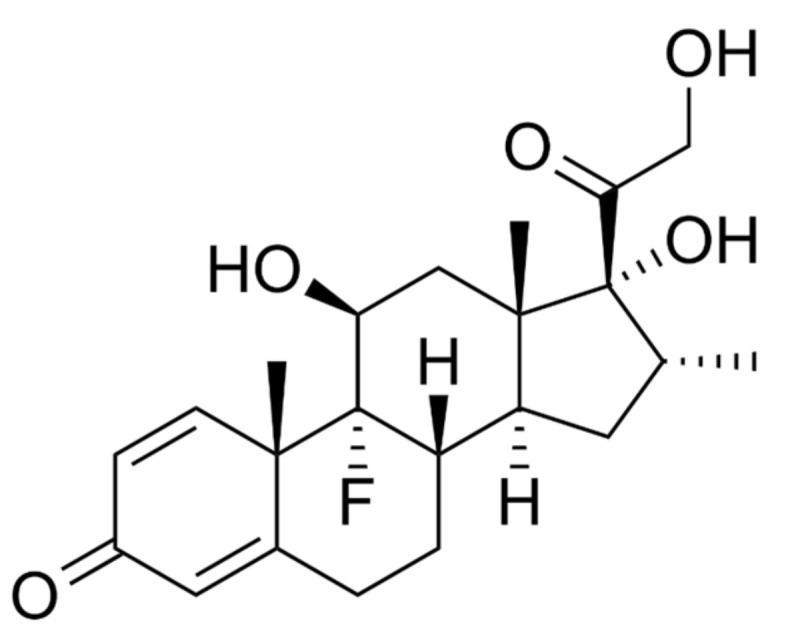
Structure of Dexamethasone.

Patients with COVID-19 who are suffering from severe hypoxemia may benefit from high doses of dexamethasone, according to recent studies [76]. Between August 2020 and May 2021, 1000 patients with COVID-19 were enrolled in a multinational, multicentred, and randomised trial [77]. Dexamethasone (Figure 17) was intravenously given to 503 patients for up to 10 days, and dexamethasone was intravenously given to 497 patients for up to 10 days. Compared to the low dose, the median number of days without life support was 22 in the dexamethasone 12 mg/day group (IQR, 6.028.0 days) [78,79]. Those who received higher doses died at a rate of 27.1% after 28 days, and those who received 6 mg of dexamethasone per day died at a rate of 32.3% (adjusted relative risk of 0.86% confidence interval of 0.681.08], After 90 days, 32% of people died, although the overall mortality rate was 37.7% (adjusted relative risk of 0.87 [99% CI 0.701.07]).11.3% and 13.4% in the 6 mg/day group, respectively; adjusted relative risk: 0.83 [99% confidence interval (CI) 0.541.29 [78,79].

#### 4.1.9. Fluvoxamine

**Figure 18:**
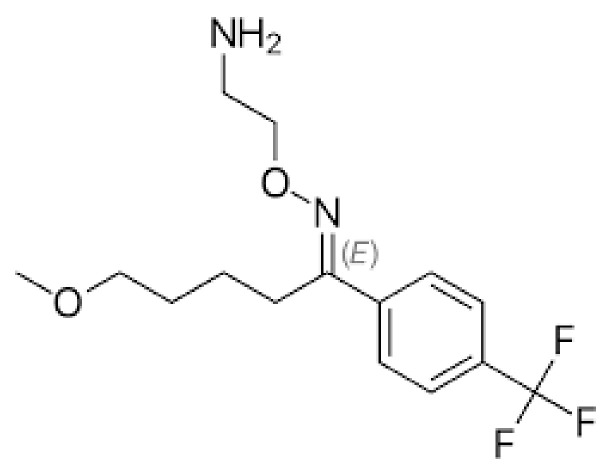
Structure of Fluvoxamine.

Fluvoxamine may reduce inflammation in rats. During the pandemic, researchers attempted to reuse fluvoxamine to treat COVID-19 [80]. In November 2020, a group of doctors published a small randomised clinical study of the effects of fluvoxamine administered to people immediately after being diagnosed with COVID-19. The large clinical study was conducted in Brazil and included 738 randomly selected COVID-19 patients and an additional 733 in a placebo group. Effective results were observed against fluvoxamine, as shown in Figure 18 [81].

### 4.2. Potential Immunotherapeutic Strategies for SARS-CoV-2

**Figure 19:**
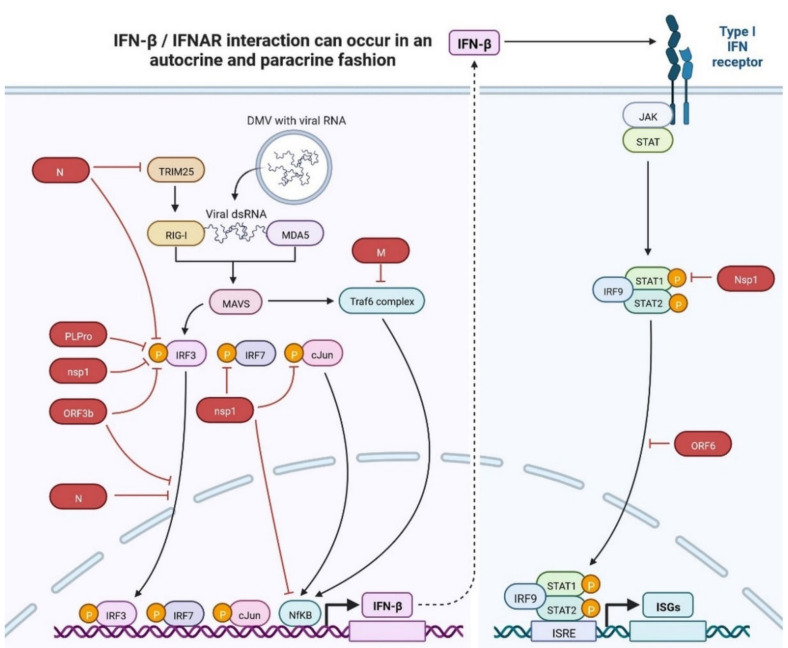
Innate immune antagonism by SARS-CoV. Notes: Red arrow suppression, black arrow activation and dotted arrow slow activation. IFN-β/IFNAR interaction can occur in an autocrine and paracrine fashion. RIG-I and MDA5 are accountable for sensing cytoplasmic viral RNAs. MAVS activates the IRF3 and supresses the regulation viral proteins. Type I interferon activates the ISGs, and the JAK-STAT signalling pathway activates the IRF9-STATcoplex, then subsequently activates the downstream kinases and transcription factors that elicit the production of IFNs and proinflammatory cytokine.

Innate immune responses are required for immunity to viral infection and act as a first line of defence mechanism [9]. The SARS-CoV-2 immune response is initiated via pattern recognition receptors (PRRs). Viral RNA is identified by extracellular and endosomal Toll-like receptors (TLRs), cytosolic RIG-I-like receptors (RLRs), cytokine production, and IFN pathways. PRRs are activated with the involvement of inhibitor-B kinases (IKKs) and TANK-binding kinase-1 (TBK1) [25,27,28,29]. These signalling molecules lead to the activation of NF-B (nuclear factor kappa-light-chain-enhancer of activated B cells) and IRF3 (interferon (IFN)-regulatory factor-3), which leads to the expression of potent cytokines, chemokines, and the anti-viral activities of IFN-stimulated genes (ISGs), type I IFNs (IFN-α/β) and proinflammatory mediators. All of this leads to the production of proinflammatory cytokines. RLRs and TLRs (Toll-like receptors) are two major receptors that activate IFN signalling and proinflammatory cytokines. Coronaviruses have developed various strategies to prevent host recognition by impeding the function of antiviral proteins using various viral proteins. IFNAR1/2 activates released type I IFNs, IFN-, and IFN- to produce the host antiviral protein. Activated forms of RLR undergo ubiquitination by E3 ligase as well as caspase activation and recruitment domains (CARD) of MAVS (mitochondrial antiviral signalling protein). This is followed by IRF (interferon regulatory factor) and NF-kB (nuclear factor kB), which results in the production of pro-inflammatory chemokines, cytokines, and type I and type III interferons (IFN/IFN). Type I interferon activates the ISGs (interferon-stimulated genes), and the JAK-STAT signalling pathway generates the anti-inflammatory cytokines and prevents the viral infection (Figure 19) [27,28,29]. In the case of SARS-CoV-2 infection, there have evolved several approaches to a form of temporary distraction from routine immune recognition that gives the necessary time or opportunity for replication of SARS-CoV-2. ZAP (Zinc finger antiviral protein) primarily interacts with CpG (cytosine-phosphate-guanosine) motifs in RNA virus genomes, and SARS-CoV-2 has evolved the most extreme cytosine approach to defend host mRNA, which prevents recognition by cytosolic PPRs [39,46]. SARS-CoV-2 has evolved a mechanism to protect the 5′ ends by cap during the replication process. This mechanism makes SARS-CoV-2 different from other viruses [39,46]. Researchers from the field found that SARS-CoV-2 produces RNA which is identifiable from cellular mRNA caps in a mouse model and leads to regulation of IFN-regulatory protein for the signalling process [46]. Another mechanism protects the viral RNA in SARS-CoV-2 by using RTC (replicase-transcriptase complex). Glycon and post-transnational modifications are also used by SARS-CoV-2 to cap immunogenic viral proteins for immune evasion [39,46]. The spike (S) protein of SARS-CoV-2 consists of two subunits, each unit composed of homotrimeric spike proteins of 8–12 nm in length with 22 glycans. These highly glycosylated proteins of SARS-CoV are regulated by DC-SIGN, which probably helps in the transmission of SARS-CoV-2 by DCs (Dendritic cells) and microphages Nsp3 protein contains DUB (de-ubiquitinating) and plays several important roles in innate immunity signalling transduction [82]. Receptor specific S proteins interact with TMRSS2 to transmit to nearby non-infected cells while evading recognition by the immune system [83]. Type I/III IFN production is inhibited by the STING-TRAF3-TBK1 complex, leading to an infection burden in the lungs in infected patients with SARS-CoV-2 [84]. SARS-CoV-2’s innate immune mechanism employs angiotensin-converting enzyme (ACE2) as a cell-receptor-binding protein and is expressed in epithelial cells of the lungs, gastrointestinal tract, cardiovascular system, and renal epithelial cells [9,27,28]. ACE2 regulates the renin–angiotensin system (RAS) by corresponding the conversion of angiotensin-I and II to angiotensin-1-9 and 1-7, respectively. After binding of SARS-CoV-2 to ACE2, endosome formation occurs, which reduces the expression level of ACE2 and induces the expression level of pro-inflammatory markers including IL-6, IL-8 (Cytokine storm), innate T cell neutrophils, and increases the production of reactive oxygen species (RAS) [85,86,87]. Researchers from the field investigated transcriptional profiles in patients with SARS-CoV-2 with the deposition of MBL, C4d, C3 and C5b-9 leading to the formation of MAC (membrane attack complex) in tissue damage with localising spike glycoprotein by antibody dependent cell medicated cytotoxicity [86]. Vascular damage and coagulation dysfunction are observed in SARS-CoV-2 patients because of the von Willebrand factor, heat shock proteins, autophagy, pyroptosis, and necroptosis [87]. The spike protein of SARS-CoV-2 also interacts with TLR-1, TLR-2, and TLR-6, causing an increase in the production of IL-8 via TLR-2 signalling in PBMC. HMGB1 (High mobility group box protein 1) signalling induces the expression of TLR-2, TLR-4, and TLR-9 in TREM-1 (triggering the receptor expressed in myeloid cell 1) [39,40,41,42,43,44,45,46]. HMGB1 is induced by MAPK (mitogen activated protein kinase) and regulates the process of cytokines in the lung tissues. HMGB1 can be a potential therapeutic option for drug targeting, which might be beneficial in pulmonary injury and sepsis in patients with SARS-CoV-2 [87,88].

#### 4.2.1. Baricitinib

Baricitinib and tocilizumab have long been used to treat inflammation and have shown effective results in immune responses [89]. Baricitinib is a Janus kinase (JAK) inhibitor. JAKs are intracellular enzymes that transmit signals which are produced when cytokines or growth factors connect with their receptors on cellular membranes to influence hemopoiesis and immune cell function [89,90]. The FDA has approved baricitinib (Olumiant tablets 1 mg and 2 mg) for the treatment of adult patients with moderately to severely active rheumatoid arthritis who have had an unsatisfactory response to one or more tumour necrosis factor antagonist therapies. At that time, the FDA had not approved baricitinib for the treatment of COVID-19. Inflammation has long been treated with baricitinib and tocilizumab, and studies have demonstrated that these medications can help reduce uncontrolled immune responses [90]. Baricitinib is an anti-inflammatory drug used for rheumatoid arthritis that reduces inflammation by inhibiting IL6 expression [89]. Tocilizumab is another arthritis drug that blocks IL6. This reduces the severity of illness and hospitalisation [84]. In July 2021, the FDA issued an emergency clearance for Olumiant (baricitinib) as a treatment of for COVID-19 in children ages 2–18 who require extracorporeal membrane oxygenation, invasive mechanical ventilation, or supplemental oxygen. On 19 November 2020, the FDA approved an EUA for the treatment of COVID-19 in a subset of hospitalised patients who need invasive mechanical ventilation, extracorporeal membrane oxygenation, or supplemental oxygen (ECMO) [90].

#### 4.2.2. Tocilizumab

Tocilizumab is another arthritis drug that blocks IL6. This reduces the severity of illness and hospitalisation [91]. The FDA approved it in July 2021 to treat inflammation and showed an effective response in severely ill patients [92]. Baricitinib is an anti-inflammatory drug used for rheumatoid arthritis that reduces inflammation by inhibiting IL6 expression [87,91,92]. The FDA approved it on 29 July 2021 based on a randomised clinical trial (NCT #4381936), and the NIH recommended baricitinib for severely hospitalised patients or patients on non-invasive ventilation [92]. Actemra was found to be effective in hospitalised adults and paediatric patients (2 years of age and older) who received corticosteroids and mechanical ventilation [92].

#### 4.2.3. Ruxolitinib

A JAK 1 and 2 inhibitors known as roxolitinib has been connected to an inhibitory effect on COVID-19 cytokines [93]. Ruxolitinib’s safety and efficacy were examined in a small, multicentre, randomised controlled phase 2 trial, which found no statistically significant differences between it and the standard of treatment [93,94]. On the other hand, most of the patients displayed a significant improvement in their chest CT scans and a quicker recovery from lymphopenia. In a significant placebo-controlled multicentre trial, ruxolitinib’s effectiveness and safety are now being assessed in patients with severe COVID-19 [95].

#### 4.2.4. Sotrovimab

The sotrovimab antibody is used to treat severely infected patients with SARS-CoV-2 [96]. In Phase 3 clinical trials, results showed a lowered risk of hospitalisation. On 26 May 2021, the FDA has approved its use for mild-to-moderate COVID-19 infected patients. The medicine was suggested on 17 November 2021 for non-hospitalized people at high risk with worsening symptoms. [97]. Sotrovimab versions B.1.1.7, B.1.351, P-1, B.1.617, and B.1.427/B.1.429/B.1.526 have also been shown to be effective. Preclinical evidence suggests that by binding to a SARS-CoV-1 and SARS-CoV-2 common epitope on SARS-CoV-2, it may both inhibit viral entry into healthy cells and eradicate infected cells [97,98].

#### 4.2.5. Casirivimab/Imdevimab

Casirivimab and imdevimab were tested in a randomised, placebo-controlled trial of outpatients with mild to moderate COVID-19 [99]. Casirivimab 600 mg in combination with imdevimab 600 mg resulted in a 2.2% reduction in overall hospitalisation and a 70% reduction in deaths. Patients who received 1200 mg IV infusions of casirivimab and imdevimab, both of which have been shown to be effective against delta variants, experienced hospitalisation, and mortality reductions of 3.3% and 71%, respectively [100].

#### 4.2.6. Tocilizumab

Tocilizumab has been demonstrated to reduce the inflammatory cascade in people with severe COVID-19 [101,102]. According to the results of a small randomized controlled trial comprising 289 patients who were randomly assigned to receive tofacitinib or a placebo, tofacitinib with steroids was effective in improving outcomes in hospitalized COVID-19 patients [101,102].

#### 4.2.7. Bebtelovimab

The neutralising IgG1 monoclonal antibody bebtelovimab recognises a particular epitope in the spike protein of the SARS-CoV-2 receptor-binding region [103]. The FDA reissued the letter of authorization on 5 August 2022 after analysing the clinical trial BLAZE-4 (NCT04634409) that assessed bebtelovimab for the treatment of mild-to-moderate COVID-19 patients [103]. It is reasonable to expect that bebtelovimab, when provided in accordance with the instructions described in the letter, may be safe and effective for treating mild-to-moderate COVID-19 patients [103,104]. For the treatment of mild-to-moderate COVID-19 in both adults and children, bebtelovimab is effective, authorised, and widely accessible [103,104].

#### 4.2.8. REGEN-COV

After approving REGEN-COV, researchers are still conducting clinical trials to better understand its efficacy. Treatment with this drug has been shown to reduce infection rates by 81% [105,106]. On 30 July 2021, FDA REGEN-COV was authorised for use in preventing COVID-19 in those who had been exposed to the virus. Preliminary results from the REGNCOV Phase 3 trial revealed a 70% reduction in hospitalisation or mortality in participants [105,106]. Two SARS-CoV-2 variants (B.1.1.7 and B.1.351) were tested in vitro for their effects on REGNCOV2, and it was discovered that REGNCOV2 retains its activity [106]. the efficacy of baricitinib alone or in combination with remdesivir have not been studied for SARS-CoV-2 mutations, and there is limited information on the use of baricitinib in combination with dexamethasone [105].

#### 4.2.9. Evusheld (Tixagevimab Co-Packaged with Cilgavimab)

The FDA issued an EUA for EVUSHELD on 8 December 2021 in COVID-19 infected patients (12 years of age or older, weighing at least 40 kg) [105,107]. The active ingredients in EVUSHELD are neutralising IgG1 monoclonal antibodies (ixagevimab and cilgavimab) that bind to certain, non-overlapping epitopes in the receptor-binding region of the SARS-CoV-2 spike protein [105,107]. The FDA issued a letter of authorization on 24 February 2022 based on a Phase III randomised, double-blind, placebo-controlled clinical trial (NCT04625725) [107].

#### 4.2.10. PD-1 Blocking Antibodies: A Potentially Repurposed Drug Candidate for COVID-19

**Figure 20:**
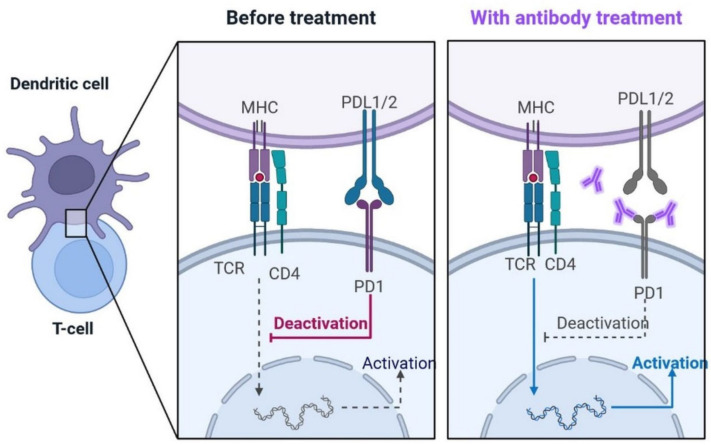
PD-1 Blocking Antibodies: Monoclonal antibodies blocking the activity of PD-1 can increase T cell proliferation, cytokine production, and reduce death in patients with severe pneumonia associated with COVID-19.

Immune checkpoint inhibitors (ICIs) are drugs that inhibit programmed death-1 (PD-1) (Figure 20). T-cell competence is restored in patients with SARS-CoV-2 infections. Anti-PD-1 and anti-PD-L1 antibody clinical trials are under investigation [108,109]. The damage caused by T cells and lymphocytopenia may lead to viral sepsis and increase the mortality rate. Anti-PD-L1-based treatment strategies may be effective options against SARS-CoV-2. Monoclonal antibodies blocking the activity of PD-1 can increase T cell proliferation and cytokine production and reduce death in patients with severe pneumonia associated with COVID-19 [108,109].

### 4.3. Sedative Drug

#### Propofol-Lipuro 1%

Propofol-Lipuro is a sedative hypnotic drug and is used intravenously in an ICU setting [110]. The FDA has reviewed the effects of Propofol-Lipuro 1% and found it effective in patients over the age of 16 and issued an approval for emergency use based on available scientific evidence [111].

### 4.4. Personalized Cell Therapies to Combat COVID-19

**Figure 21:**
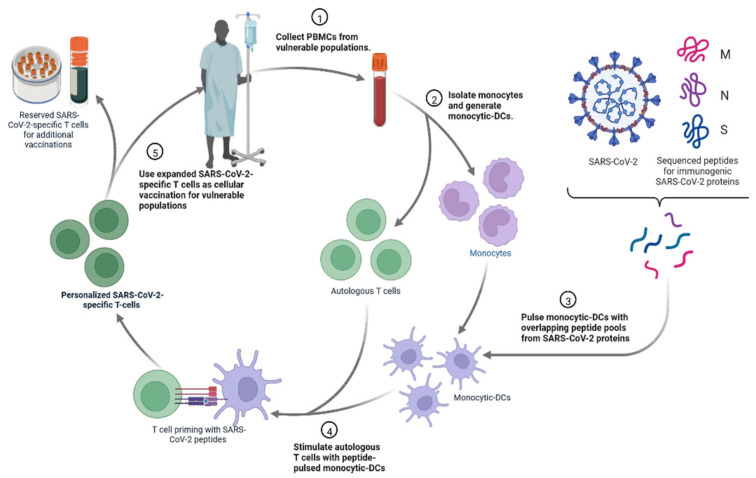
The personalised viral-specific T cells are used as a potential therapeutic to prevent and/or treat SARS-CoV-2 infections.

The personalised viral-specific T cells are used as a potential therapeutic to prevent and/or treat SARS-CoV-2 infections among vulnerable populations, such as cancer patients (Figure 21) [112,113]. An individual’s own monocytic-DCs are pulsed with SARS-CoV-2 peptides and then used to prime that same individual’s T cells to generate SARS-CoV-2-specific T cells [110,111]. These T cells could be cryopreserved or infused into a vulnerable individual as a COVID-19 prophylactic or treatment [112].

### 4.5. Gene Therapy

**Figure 22:**
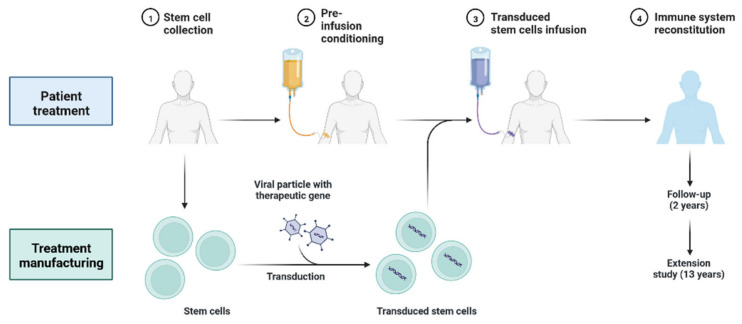
Gene Therapy Treatment Phases.

Gene therapy products aim to change a gene’s expression or the genetic activities of living cells for therapeutic purposes during the treating phases of gene therapy (Figure 22). Cellular immunotherapies, as well as a variety of autologous and allogeneic cell types, are products used in cell therapy [112].

### 4.6. Other Approaches for Drug Development

#### 4.6.1. Recombinant ACE-2 Approaches

**Figure 23:**
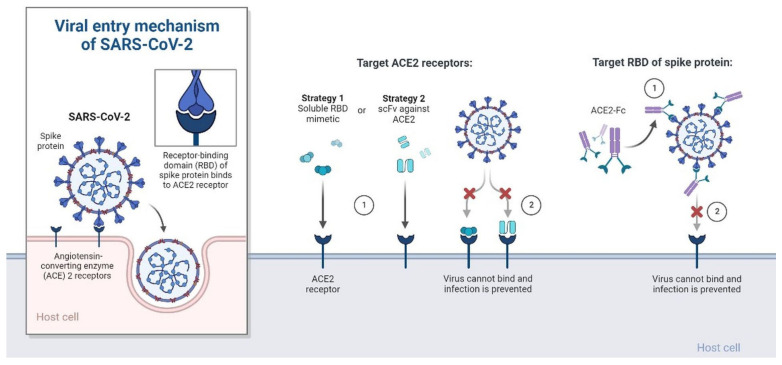
Therapeutic strategies to treat SARS-CoV-2 infection based on virus-cell interaction. Host-targeted strategies include RBD mimetics and antibody fragments, such as scFv. Virally targeted strategies include antibodies or antibody fragments, such as Fc. In both cases, the ACE2-RBD interaction is inhibited, preventing infection. The template can be either used as is or adapted to demonstrate other virus-cell interactions.

Angiotensin-converting enzyme 2 (ACE2), sometimes referred to as ACEH (ACE homolog), is a zinc metalloprotease that is a member of the ACE family of enzymes [113]. The predicted human ACE2 protein sequence consists of an N-terminal signal peptide, a single catalytic domain, a C-terminal membrane anchor, and a short cytoplasmic tail [114]. Angiotensin I and II are cleaved by ACE2 as a carboxypeptidase (Figure 23) [115]. ACE2 is a critical negative regulator of the RAS (renin–angiotensin system). RAS signalling pathways and ACE2 appear to play essential roles in SARS-CoV-induced acute respiratory distress syndrome (ARDS) and deadly avian influenza A (H5N1, H7N9)-induced acute lung damage, according to new research (ALI) [116,117]. Furthermore, an overabundance of rhACE2 can disrupt the RAS equilibrium. To avoid the underlying adverse effects associated with RAS, it is critical to select an effective injection volume. The chimeric fusion of rhACE2 with an IgG2-Fc fragment has recently been demonstrated to improve rhACE2 plasma stability [48]. In mice, the rhACE2Fc fusion protein kept its complete peptidase function and had a longer plasma half-life [116]. The rhACE2Fc method is projected to improve patient comfort, reduce dose frequency, and improve treatment efficacy significantly [117]. According to pathological results, SARS-CoV-2 is also linked to lung failure and ARDS [117,118]. The in vitro neutralising effects that were observed showed that human ACE2 fused with the Fc region of human IgG1, and the plant produced ACE2 Fc fusion protein in Nicotiana *benthamiana* [119]. Six days after infiltration, the recombinant ACE2Fc fusion protein was produced in Nicotiana benthamiana leaves with a fresh weight of 100 g/mL [119,120]. The SARS-receptor CoV-2’s binding domain (RBD) was strongly bound by the recombinant fusion protein. In vitro, it is critical that plant-produced fusion proteins have high anti-SARS-CoV-2 activity [119,120]. After viral infection, Vero cells are treated with the ACE2 Fc fusion protein, which significantly reduces SARS-CoV-2 infectivity. The IC50 value is 0.84 g/mL. Pre-entry therapy with the ACE2 Fc fusion protein also inhibited SARS-CoV-2 infection, with an IC50 of 94.66 g/mL [119,120]. These findings point to plant-produced ACE2 Fc fusion proteins as potential SARS-CoV-2 treatment options. In cellular and animal experiments, recombinant ACE2 proteins showed encouraging outcomes for SARS-CoV-2, and preliminary clinical studies suggested that they are safe for humans [119,120]. Their efficacy is currently being investigated in large-scale investigations. ACE2 has a strong affinity for the S RBD mutation and has been demonstrated to be effective against it; it also has an affinity for both the E484K and the N501Y mutations [98].

#### 4.6.2. Renal Replacement Therapies (CRRT)

SARS-CoV-2 increased the frequency of severe infections and multiple organ failure, particularly acute renal injury, which increased the need for CRRT [121]. The prevalence of multiple organ failure, including acute kidney injury, has increased, to making CRRT necessary in COVID-19 patients [122]. The FDA has given approval based on available scientific data for CRRT in a critical care situation.

#### 4.6.3. Cell-Free Biotherapeutic

Zofin is a cell-free biotherapeutic agent derived from naturally occurring microRNAs [123,124]. The first 10 patients in the study were those with moderate to severe COVID-19. They were all successfully treated in hospitals in Bangalore, Calicut, and Chennai, and were able to return home. Another 65 patients with moderate to severe COVID-19 were enrolled in a research trial this year thanks to a partnership between Organic ell and CWI India [124]. There have been documented instances of Zofin being used for compassionate purposes in the United States, and Organicell recently reported using Zofin for patients who have mild to intermediate COVID-19 or who are at high risk of developing moderate COVID-19. The extracellular microvesicles/nanoparticles from perinatal tissue contain more than 300 growth factors, cytokines, and chemokines [124]. The safety and efficacy of intravenous injections of Zofin TM and placebo in the treatment of moderate to SARS-related COVID-19 infections are currently being evaluated in a Phase I/II randomised, double-blind, placebo study. B.1.1.7, P.1, B.1.617, B.1.427/B.1.429, and B.1.526 have all been shown to be vulnerable to Zofin [98].

## 5. Nanomaterials for the Treatment of COVID-19

**Figure 24:**
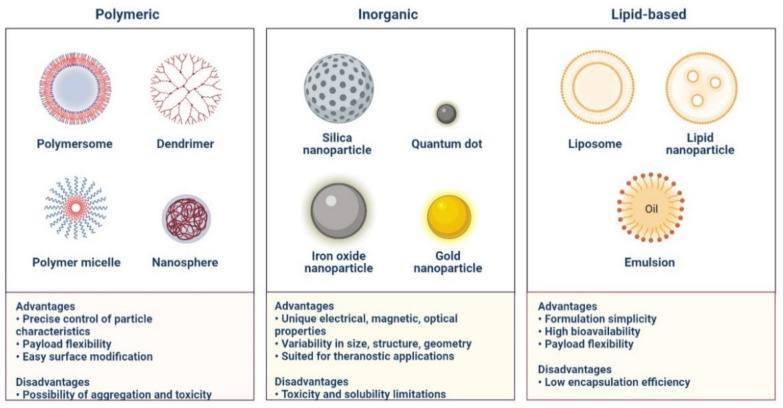
Classes of Nanoparticles.

Nanomaterials have been used to successfully neutralise viruses like the SARS and MERS coronaviruses (Figure 24) [125]. The creation of novel anti-SARS-CoV-2 therapeutics using nanomaterials has shown potential [126]. The S protein of SARS-CoV-2 and ACE2 on the host cell membrane have been used to create a medication for SARS-CoV-2 [124]. The PIH/Au nanomaterial prevents the virus from entering the cells. To block virus entry, boronic acid ligands and carbon quantum dots (CQDs) interfere with the interaction between the protein S-receptor and the host cell membrane [125,126]. Silver nanoparticles (Ag NPs) were used as a first line of defence to stop SARS-CoV-2 infection.

RNA viruses are prevented from integrating into host cells by the AgNPs, which prevent Ag NPs from adhering to the surface glycoproteins of RNA viruses [127]. The SARS-CoV-2 virus has been demonstrated to be sensitive to copper. Graphene oxide treated with amino groups served as the signalling pathway for STAT1/IRF1 interferon in T cells, mediating the induction of T cell chemo-attractant. The development of next-generation vaccines is made possible by nanomaterials that mimic the inherent immunostimulatory capabilities of viruses [127,128]. A messenger RNA (mRNA) lipid nanoparticle vaccine has been investigated to fight SARS-CoV and MERS. It has been demonstrated that liposomes, dendrimers, micelles, and polymer-based nanoparticles have anti-SARS-CoV-2 characteristics [128,129].

**Figure 25:**
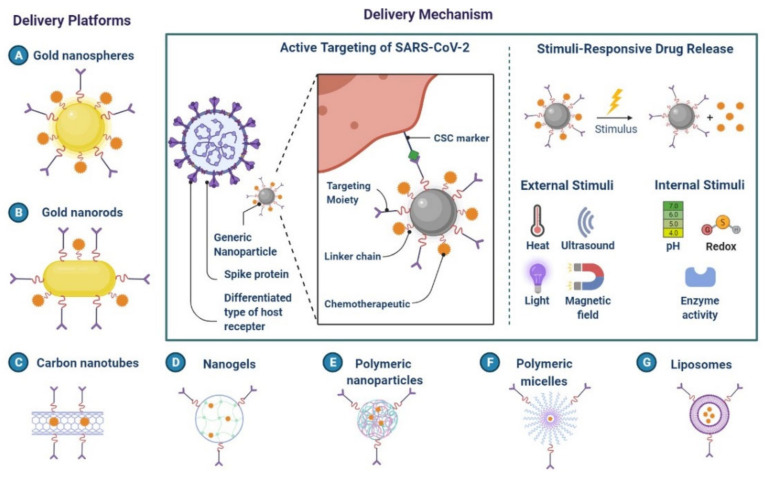
Nanoparticle-Mediated Targeted Drug Delivery.

**Figure 26:**
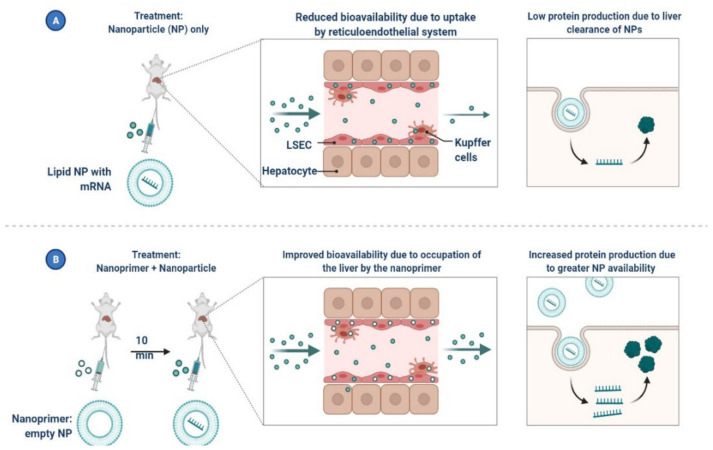
A Nanoprimer to Improve the Systemic Delivery of mRNA-Containing Nanoparticles.

Nano-drug co-delivery can reduce particle size-dependent safety concerns in the respiratory and pulmonary systems. For effective and secure pulmonary delivery, corticosteroid-loaded PLGA NPs, solid lipid NPs, N, N-dimethylaminoethyl methacrylate, and butyl methacrylate monomers are used (Figure 24 and Figure 25) [128,129]. Mesoporous silica NPs are functionalized with ligands for active viral cell targeting, enabling drug co-delivery [127]. AgNPs are more effective drug carriers for nucleic acid-based delivery because of their improved stability, resistance to degradation, and controlled intracellular delivery, shown in A Nanoprimer to Improve the Systemic Delivery of mRNA-Containing Nanoparticles (Figure 26) [129,130]. Theaflavin and AgNPs have been shown to inhibit viral replication and lessen the severity of COVID-19 symptoms [129]. It is possible to use cytokine and antibody responses that are induced by AuNPs, carbon-based NPs, polymeric NPs, and vesicular nanocarriers to deliver antigens to certain sites [131,132].

Nano-sized herbal remedies have been developed as nano-phytomedicines for alternative and complementary medicine based on their properties, which may help in the fight against SARS-CoV-2 [127,132,133,134]. Numerous conventional treatments have been used to stop the viral life cycle since the COVID-19 pandemic began. Some accounts state that, during the COVID-19 outbreak, more Chinese, Indonesian, and Nepalese individuals used medicinal herbs because they believed the herbs could effectively prevent or treat the disease [133]. Traditional medications combined with nanomedicine can be very helpful in the fight against SASR-CoV-2 [134]. Secondary metabolites of plants that can be delivered by spherical nanoparticles. Glycyrrhizic acid has a known anti-SARS-CoV effect because of its cytotoxicity, poor water and bio-fluid solubility, and low bioavailability [135].

## 6. Conclusions

This study shows the limitations of existing SARS-CoV-2 drug discovery efforts and a lack of knowledge of the regulatory mechanisms governing emerging COVID-19 sub-types. In the future, emerging variants should be considered to close the gap in the fight against SARS-CoV-2. Alternative or innovative therapeutic strategies are urgently required for the treatment of COVID-19. Since host factors are crucial regulators of SARS-CoV-2 infection, they are potential targets for antiviral treatment. Therefore, identifying novel host genes or proteins that mediate COVID-19 pathogenesis, along with associated signalling pathways, is an essential tool that could aid in our understanding of the precise biological pathogenesis and, by approach, identify host-directed treatment targets for SARS-CoV-2 infection.

## Figures and Tables

**Table 1:** Comparison of the epidemiological, clinical, and radiological characteristics of SARS-CoV, MERS-CoV and SARS-CoV-2 diseases.

Characteristics	SARS-CoV-2	MERS-CoV	SARS-CoV
Outbreak Beginning Date	December 2019 [18]	April 2012 [20]	November 2002 [20]
Location of the First Case	Wuhan, China [18]	Saudi Arabia [20]	Guangdong, China [20]
Confirmed Cases	651,918,402 [From December 2019 to 23 December 2022] [22]	2519 (From 2012 until 31 January 2020) [20]	8096 [20]
Latency	Between 5 and 14 days [18,19]	Between 2 and 14 days [20]	Between 2 and 7 days [20]
Contagious Period	5 to 20 days after onset of disease, depends on severity of disease	5–14 days after onset of disease [20]	10 days after onset of disease [20]
Fatality Rate	~2.3% [19]	~36% [20]	~10% [20]
Incidental Host	Malayan pangolin [19,20]	Dromedary camels [20]	Masked palm civets [20]
Transmission	Respiratory droplets Possibly faecal-oral Close contact with diseased patients Possibly aerosol [18,19]	Respiratory droplets Ingestion of camel milk Close contact with diseased patients/camels [20]	Respiratory droplets Faecal-oral Close contact with diseased patients Aerosol [20]
Radiologic Features	Various, from focal faint patchy ground-glass opacity to bilateral ambiguous consolidation of the air space of plain chest radiographs. Not sufficiently unique to differentiate between the three diseases [20].		
Clinical Presentation	From asymptomatic or moderate illness to severe upper-respiratory distress and death-related multiorgan failure, varying from person to person. Diarrhoea is recorded as well [20].		

**Table 2:** SARS-CoV-2 variants and their effects on clinical outcome.

Lineage	Strain	Mutations (Spike Protein)	First Identified	Effects on Clinical Outcome	References
B.1.1.7	20I/501Y.V1	N501Y, A570D, D614G, P681H, Δ69/70, Δ144Y.	United Kingdom	Increased severity based on hospitalizations and case fatality rates Limited effect of monoclonal antibody treatments on neutralising	[10,33]
P.1	20J/501Y.V3	K417N/T E484K N501Y D614G	Japan/Brazil	Significant effect of some monoclonal antibody therapy on neutralising Bamlanivimab–etesevimab: reduced susceptibility. Casirivimab-imdevimab: No change in susceptibility	[33,35]
B.1.351	20H/501.V2	N501Y D614G K417N E484K	South Africa	Increased transmission (50%). Bamlanivimab–etesevimab: >45-fold decrease in susceptibility	[35]
B.1.427 and B.1.429	20C/S:452R	S13I W152C L452R D614G	US-California	Increased transmission (20%). Bamlanivimab–etesevimab: Active but 7.4-fold reduced the susceptibility	[33]

**Table 3:** Variants of Concern.

Name of Emerged Variants	Lineage	Name of Country (First Identified)	Mutation of Interest in Spike Region	Year and Month First Detected	Effect on Disease Severity	Rate of Transmission	References
Omicron	BA.2	South Africa	T376A, D405N, R408S, K417N, N440K, S477N, T478K, E484A, Q954H, N969K, G142D, N211I, Δ212, V213G, G339D, S371F, S373P, S375F, Q493R, Q498R, N501Y, Y505H, D614G, H655Y, N679K, P681H, N764K, D796Y	In November 2021	High	Community level	[32]
Omicron	BA.4	South Africa	L452R, F486V, R493Q	In January 2022	High	Community level	[38]
Omicron	BA.5	South Africa	L452R, F486V, R493Q	In February 2022	High	Community level	[37]

**Table 4:** Variants of Interest.

Name of Emerged Variants	Lineage	Name of Country (First Identified)	Mutation of Interest in Spike Region	Year and Month First Detected	Effect on Disease Severity	Rate of Transmission	References
Omicron	BA.2.75	India	W152R, F157L, I210V, G257S, D339H, G446S, N460K, Q493 (reversion)	In May 2022	High	Not Known	[32]
Omicron	BQ.1	No evidence	K444T, N460K	No evidence	High	No evidence	[36]

**Table 5:** Variants under monitoring.

Name of Emerged Variants	Lineage	Name of Country (First Identified)	Mutation of Interest in Spike Region	Year and Month First Detected	Effect on Disease Severity	Rate of Transmission	References
Omicron	B.1.1.529+R346X Any amino-acid substitution	No evidence	R346X	No evidence		No evidence	[33]
Omicron	B.1.1.529+K444X, N460X	No evidence	K444X, N460X	No evidence	High	No evidence	[37]
Omicron	B.1.1.529+N460X, F490X, including XBB and other sub-lineages	No evidence	N460X, F490X	No evidence	High	No evidence	[38]

**Table 6:** List of drugs used in SARS-CoV-2 treatment.

Name of the Drug	Potential	Description	References
Remdesivir	Used as antiviral, inhibiting RNA synthesis in coronaviruses	Selectively provided in 2020 for COVID-19 emergency use, both promising and negative effects reported; Trial was sponsored by Gilead, WHO, INSERM, NIAID.	[49,50,51]
Hydroxychloroquine or chloroquine	Used for malaria, lupus (international), rheumatoid arthritis	Selectively provided in 2020 for COVID-19 emergency use, both promising and negative effects reported; Trial was sponsored by CEPI, WHO, INSERM; Discontinued by WHO.	[52]
Favipiravir	Used as antiviral against influenza	Used against COVID-19 in 2020; Favipiravir reduced mortality, but the finding was not statistically significant; Trial was sponsored by Fujifilm, China.	[53]
Lopinavir/ritonavir without or with interferon beta-1a	Used as antiviral, immune suppression.	Ritonavir has been proposed as a treatment for COVID-19 based on in vitro activity, preclinical studies, and observational studies; Trial was sponsored by CEPI, WHO, UK Government, Univ. of Oxford, INSERM	[54]
Sarilumab	Human monoclonal antibody against interleukin-6 receptor	Clinical efficacy of sarilumab relative to the control arm in adult participants hospitalized with severe or critical coronavirus disease 2019; Clinical trial sponsored by Regeneron-Sanofi.	[55]
Ritonavir+ ASC-09	Antiviral	Clinical trial determined whether PF-07321332/ritonavir is safe and effective for the treatment of adults; Trial was performed at multiple sites in China; Combination not approved; ritonavir approved for HIV.	[56]
Tocilizumab	Used in rheumatoid arthritis, immune suppression (US, Europe).	Ritonavir has been proposed as a treatment for COVID-19 based on Phase III trial and sponsored by Tocilizumab Genentech-Hoffmann-La Roche	[57]
Lenzilumab	New drug candidate; Humanized monoclonal antibody for relieving pneumonia	Tested against COVID-19 in 2020; Trial was sponsored by Humanigen, Inc.	[58]
Dapagliflozin	Used as hypoglycaemia agent	Dapagliflozin did not significantly reduce the severity of infection; Trial was sponsored in 2020 by Saint Luke’s Mid America Heart Institute, AstraZeneca.	[59]
CD24Fc	Used as antiviral immunomodulator against inflammatory response	Phase III study to evaluate the safety and efficacy of CD24Fc in COVID-19 treatment; Trial was sponsored by OncoImmune, Inc. in 2021	[60]
Apabetalone	Selective BET inhibitor	Tested against COVID-19 in 2020; Trial was sponsored by Resverlogix Corp	[61]

## Data Availability

Not applicable.

## References

[1] Abouelela M.E., Assaf H.K., Abdelhamid R.A., Elkhyat E.S., Sayed A.M., Oszako T., Belbahri L., El Zowalaty A.E., Abdelkader M.S.A. (2021). Identification of Potential SARS-CoV-2 Main Protease and Spike Protein Inhibitors from the Genus *Aloe*: An In Silico Study for Drug Development. Molecules.

[2] Overhoff B., Falls Z., Mangione W., Samudrala R. (2021). A Deep-Learning Proteomic-Scale Approach for Drug Design. Pharmaceuticals.

[3] Meng J., Li R., Zhang Z., Wang J., Huang Q., Nie D., Fan K., Guo W., Zhao Z., Han Z. (2022). A Review of Potential Therapeutic Strategies for COVID-19. Viruses.

[4] Starshinova A., Malkova A., Zinchenko U., Kudlay D., Glushkova A., Dovgalyk I., Yablonskiy P., Shoenfeld Y. (2021). Efficacy of Different Types of Therapy for COVID-19: A Comprehensive Review. Life.

[5] Gherghescu I., Delgado-Charro M.B. (2021). The Biosimilar Landscape: An Overview of Regulatory Approvals by the EMA and FDA. Pharmaceutics.

[6] Bauso L.V., Imbesi C., Irene G., Calì G., Bitto A. (2021). New Approaches and Repurposed Antiviral Drugs for the Treatment of the SARS-CoV-2 Infection. Pharmaceuticals.

[7] D’Alessandro S., Scaccabarozzi D., Signorini L., Perego F., Ilboudo D.P., Ferrante P., Delbue S. (2020). The Use of Antimalarial Drugs against Viral Infection. Microorganisms.

[8] FDA Drug Competition Action Plan. https://www.fda.gov/drugs/guidance-compliance-regulatory-information/fda-drug-competition-action-plan.

[9] Queirós-Reis L., Gomes da Silva P., Gonçalves J., Brancale A., Bassetto M., Mesquita J.R. (2021). SARS-CoV-2 Virus−Host Interaction: Currently Available Structures and Implications of Variant Emergence on Infectivity and Immune Response. Int. J. Mol. Sci..

[10] Ramesh S., Govindarajulu M., Parise R.S., Neel L., Shankar T., Patel S., Lowery P., Smith F., Dhanasekaran M., Moore T. (2021). Emerging SARS-CoV-2 Variants: A Review of Its Mutations, Its Implications and Vaccine Efficacy. Vaccines.

[11] Yang L., Li J., Guo S., Hou C., Liao C., Shi L., Ma X., Jiang S., Zheng B., Fang Y. (2021). SARS-CoV-2 Variants, RBD Mutations, Binding Affinity, and Antibody Escape. Int. J. Mol. Sci..

[12] Majeed A., Lee S. (2021). Applications of Machine Learning and High-Performance Computing in the Era of COVID-19. Appl. Syst. Innov..

[13] Peyclit L., Yousfi H., Rolain J.-M., Bittar F. (2021). Drug Repurposing in Medical Mycology: Identification of Compounds as Potential Antifungals to Overcome the Emergence of Multidrug-Resistant Fungi. Pharmaceuticals.

[14] Zhuang D., Ibrahim A.K. (2021). Deep Learning for Drug Discovery: A Study of Identifying High Efficacy Drug Compounds Using a Cascade Transfer Learning Approach. Appl. Sci..

[15] Agrawal L., Poullikkas T., Eisenhower S., Monsanto C., Bakku R.K., Chen M.-H., Kalra R.S. (2021). Viroinformatics-Based Analysis of SARS-CoV-2 Core Proteins for Potential Therapeutic Targets. Antibodies.

[16] Miyazaki M., Yanagida R., Nakashima A., Matsuo K., Moriwaki N., Uchiyama M., Yamada Y., Hirata H., Kushima H., Kinoshita Y. (2022). Evaluation of Remdesivir for Mildly to Moderately Ill Patients with COVID-19: A Single-Arm, Single-Center, Retrospective Study. Medicina.

[17] Magazine N., Zhang T., Wu Y., McGee M.C., Veggiani G., Huang W. (2022). Mutations and Evolution of the SARS-CoV-2 Spike Protein. Viruses.

[18] Fernández-De-Las-Peñas C., Notarte K.I., Peligro P.J., Velasco J.V., Ocampo M.J., Henry B.M., Arendt-Nielsen L., Torres-Macho J., Plaza-Manzano G. (2022). Long-COVID Symptoms in Individuals Infected with Different SARS-CoV-2 Variants of Concern: A Systematic Review of the Literature. Viruses.

[19] Ramadori G.P. (2022). SARS-CoV-2-Infection (COVID-19): Clinical Course, Viral Acute Respiratory Distress Syndrome (ARDS) and Cause(s) of Death. Med Sci..

[20] Pustake M., Tambolkar I., Giri P., Gandhi C. (2022). SARS, MERS and COVID-19: An overview and comparison of clinical, laboratory and radiological features. J. Fam. Med. Prim. Care.

[21] Jiang W., Ji W., Zhang Y., Xie Y., Chen S., Jin Y., Duan G. (2022). An Update on Detection Technologies for SARS-CoV-2 Variants of Concern. Viruses.

[22] World Health Organization (WHO) Coronavirus COVID-19 Dashboard. https://covid19.who.int.

[23] Kaivola J., Nyman T.A., Matikainen S. (2021). Inflammasomes and SARS-CoV-2 Infection. Viruses.

[24] Di Sante G., Buonsenso D., De Rose C., Tredicine M., Palucci I., De Maio F., Camponeschi C., Bonadia N., Biasucci D., Pata D. (2022). Immunopathology of SARS-CoV-2 Infection: A Focus on T Regulatory and B Cell Responses in Children Compared with Adults. Children.

[25] Rabaan A.A., Al-Ahmed S.H., Muhammad J., Khan A., Sule A.A., Tirupathi R., Mutair A.A., Alhumaid S., Al-Omari A., Dhawan M. (2021). Role of Inflammatory Cytokines in COVID-19 Patients: A Review on Molecular Mechanisms, Immune Functions, Immunopathology and Immunomodulatory Drugs to Counter Cytokine Storm. Vaccines.

[26] Liu Q., Chi S., Dmytruk K., Dmytruk O., Tan S. (2022). Coronaviral Infection and Interferon Response: The Virus-Host Arms Race and COVID-19. Viruses.

[27] Howard F.H.N., Kwan A., Winder N., Mughal A., Collado-Rojas C., Muthana M. (2022). Understanding Immune Responses to Viruses—Do Underlying Th1/Th2 Cell Biases Predict Outcome?. Viruses.

[28] Reed S.G., Ager A. (2022). Immune Responses to IAV Infection and the Roles of L-Selectin and ADAM17 in Lymphocyte Homing. Pathogens.

[29] Rubio-Casillas A., Redwan E.M., Uversky V.N. (2022). SARS-CoV-2: A Master of Immune Evasion. Biomedicines.

[30] Lim H.X., Masomian M., Khalid K., Kumar A.U., MacAry P.A., Poh C.L. (2022). Identification of B-Cell Epitopes for Eliciting Neutralizing Antibodies against the SARS-CoV-2 Spike Protein through Bioinformatics and Monoclonal Antibody Targeting. Int. J. Mol. Sci..

[31] Morales-Núñez J.J., Muñoz-Valle J.F., Torres-Hernández P.C., Hernández-Bello J. (2021). Overview of Neutralizing Antibodies and Their Potential in COVID-19. Vaccines.

[32] Kherabi Y., Launay O., Luong Nguyen L.B. (2022). COVID-19 Vaccines against Omicron Variant: Real-World Data on Effectiveness. Viruses.

[33] Redwan E.M., Elrashdy F., Aljabali A.A.A., Baetas-da-Cruz W., Barh D., Brufsky A.M., Hassan S.S., Lundstrom K., Serrano-Aroca Á., Takayama K. (2022). Would New SARS-CoV-2 Variants Change the War against COVID-19?. Epidemiologia.

[34] Berno G., Fabeni L., Matusali G., Gruber C.E.M., Rueca M., Giombini E., Garbuglia A.R. (2022). SARS-CoV-2 Variants Identification: Overview of Molecular Existing Methods. Pathogens.

[35] Lee C., Mangalaganesh S., Wilson L.O.W., Kuiper M.J., Drew T.W., Vasan S.S. (2022). Tracking Co-Occurrence of N501Y, P681R, and Other Key Mutations in SARS-CoV-2 Spike for Surveillance. Zoonotic Dis..

[36] Emmelot M.E., Vos M., Boer M.C., Rots N.Y., de Wit J., van Els C.A.C.M., Kaaijk P. (2022). Omicron BA.1 Mutations in SARS-CoV-2 Spike Lead to Reduced T-Cell Response in Vaccinated and Convalescent Individuals. Viruses.

[37] Basile K., Rockett R.J., McPhie K., Fennell M., Johnson-Mackinnon J., Agius J.E., Fong W., Rahman H., Ko D., Donavan L. (2022). Improved Neutralisation of the SARS-CoV-2 Omicron Variant following a Booster Dose of Pfizer-BioNTech (BNT162b2) COVID-19 Vaccine. Viruses.

[38] Kandeel M., Mohamed M.E.M., Abd El-Lateef H.M.A., Venugopala K.N., El-Beltagi H.S. (2022). Omicron variant genome evolution and phylogenetics. J. Med Virol..

[39] Bellamkonda N., Lambe U.P., Sawant S., Nandi S.S., Chakraborty C., Shukla D. (2022). Immune Response to SARS-CoV-2 Vaccines. Biomedicines.

[40] Ng T.I., Correia I., Seagal J., DeGoey D.A., Schrimpf M.R., Hardee D.J., Noey E.L., Kati W.M. (2022). Antiviral Drug Discovery for the Treatment of COVID-19 Infections. Viruses.

[41] Heustess A.M., Allard M.A., Thompson D.K., Fasinu P.S. (2021). Clinical Management of COVID-19: A Review of Pharmacological Treatment Options. Pharmaceuticals.

[42] Focosi D., Franchini M., Pirofski L.-A., Burnouf T., Fairweather D., Joyner M.J., Casadevall A. (2021). COVID-19 Convalescent Plasma Is More than Neutralizing Antibodies: A Narrative Review of Potential Beneficial and Detrimental Co-Factors. Viruses.

[43] Kim K., Bae K.S., Kim H.S., Lee W.-Y. (2022). Effectiveness of Mesenchymal Stem Cell Therapy for COVID-19-Induced ARDS Patients: A Case Report. Medicina.

[44] Almagro J.C., Mellado-Sánchez G., Pedraza-Escalona M., Pérez-Tapia S.M. (2022). Evolution of Anti-SARS-CoV-2 Therapeutic Antibodies. Int. J. Mol. Sci..

[45] Castro e Silva A., Bernardes A.T., Barbosa E.A.G., das Chagas I.A.S., Dáttilo W., Reis A.B., Ribeiro S.P. (2022). Successive Pandemic Waves with Different Virulent Strains and the Effects of Vaccination for SARS-CoV-2. Vaccines.

[46] Voulgaridi I., Sarrou S., Dadouli A., Peristeri A.-M., Nasika A., Onoufriadis I., Kyritsi M.A., Anagnostopoulos L., Theodoridou A., Avakian I. (2022). Intensity of Humoral Immune Responses, Adverse Reactions, and Post-Vaccination Morbidity after Adenovirus Vector-Based and mRNA Anti-COVID-19 Vaccines. Vaccines.

[47] Hsu J.-Y., Mao Y.-C., Liu P.-Y., Lai K.-L. (2021). Pharmacology and Adverse Events of Emergency-Use Authorized Medication in Moderate to Severe COVID-19. Pharmaceuticals.

[48] Esposito R., Mirra D., Sportiello L., Spaziano G., D’Agostino B. (2022). Overview of Antiviral Drug Therapy for COVID-19: Where Do We Stand?. Biomedicines.

[49] Terkes V., Lisica K., Marusic M., Verunica N., Tolic A., Morovic M. (2022). Remdesivir Treatment in Moderately Ill COVID-19 Patients: A Retrospective Single Center Study. J. Clin. Med..

[50] Al-Tannak N.F., Novotny L., Alhunayan A. (2020). Remdesivir—Bringing Hope for COVID-19 Treatment. Sci. Pharm..

[51] Ceramella J., Iacopetta D., Sinicropi M.S., Andreu I., Mariconda A., Saturnino C., Giuzio F., Longo P., Aquaro S., Catalano A. (2022). Drugs for COVID-19: An Update. Molecules.

[52] Taieb F., Mbaye K.D., Tall B., Lakhe N.A., Talla C., Thioub D., Ndoye A.M., Ka D., Gaye A., Cissé Diallo V.M.-P. (2021). Hydroxychloroquine and Azithromycin Treatment of Hospitalized Patients Infected with SARS-CoV-2 in Senegal from March to October. J. Clin. Med..

[53] McAleer M. (2020). Prevention Is Better Than the Cure: Risk Management of COVID-19. J. Risk Financial Manag..

[54] Rattanaumpawan P., Jirajariyavej S., Lerdlamyong K., Palavutitotai N., Saiyarin J. (2022). Real-World Effectiveness and Optimal Dosage of Favipiravir for Treatment of COVID-19: Results from a Multicenter Observational Study in Thailand. Antibiotics.

[55] Gentile I., Scotto R., Schiano Moriello N., Pinchera B., Villari R., Trucillo E., Ametrano L., Fusco L., Castaldo G., Buonomo A.R. (2022). Nirmatrelvir/Ritonavir and Molnupiravir in the Treatment of Mild/Moderate COVID-19: Results of a Real-Life Study. Vaccines.

[56] Marino A., Munafò A., Augello E., Bellanca C.M., Bonomo C., Ceccarelli M., Musso N., Cantarella G., Cacopardo B., Bernardini R. (2022). Sarilumab Administration in COVID-19 Patients: Literature Review and Considerations. Infect. Dis. Rep..

[57] Maraolo A.E., Crispo A., Piezzo M., Di Gennaro P., Vitale M.G., Mallardo D., Ametrano L., Celentano E., Cuomo A., Ascierto P.A. (2021). The Use of Tocilizumab in Patients with COVID-19: A Systematic Review, Meta-Analysis and Trial Sequential Analysis of Randomized Controlled Studies. J. Clin. Med..

[58] Hussen J., Kandeel M., Hemida M.G., Al-Mubarak A.I.A. (2020). Antibody-Based Immunotherapeutic Strategies for COVID-19. Pathogens.

[59] Temesgen Z., Burger C.D., Baker J., Polk C., Libertin C.R., Kelley C.F., Marconi V.C., Orenstein R., Catterson V.M., Aronstein W.S. (2022). Lenzilumab in hospitalised patients with COVID-19 pneumonia (LIVE-AIR): A phase 3, randomised, placebo-controlled trial. Lancet Respir. Med..

[60] Ho C., Lee P.-C. (2022). COVID-19 Treatment—Current Status, Advances, and Gap. Pathogens.

[61] Welker J., Pulido J.D., Catanzaro A.T., Malvestutto C.D., Li Z., Cohen J.B., Whitman E.D., Byrne D., Giddings O.K., Lake J.E. (2022). Efficacy and safety of CD24Fc in hospitalised patients with COVID-19: A randomised, double-blind, placebo-controlled, phase 3 study. Lancet Infect. Dis..

[62] Gilham D., Smith A.L., Fu L., Moore D.Y., Muralidharan A., Reid S.P.M., Stotz S.C., Johansson J.O., Sweeney M., Wong N.C.W. (2021). Bromodomain and Extraterminal Protein Inhibitor, Apabetalone (RVX-208), Reduces ACE2 Expression and Attenuates SARS-CoV-2 Infection In Vitro. Biomedicines.

[63] Wicik Z., Eyileten C., Jakubik D., Simões S.N., Martins D.C., Pavão R., Siller-Matula J.M., Postula M. (2020). ACE2 Interaction Networks in COVID-19: A Physiological Framework for Prediction of Outcome in Patients with Cardiovascular Risk Factors. J. Clin. Med..

[64] Lee C.-C., Hsieh C.-C., Ko W.-C. (2021). Molnupiravir—A Novel Oral Anti-SARS-CoV-2 Agent. Antibiotics.

[65] Jayk Bernal A., Gomes da Silva M.M., Musungaie D.B., Kovalchuk E., Gonzalez A., Delos Reyes V., Martín-Quirós A., Caraco Y., Williams-Diaz A., Brown M.L. (2022). Molnupiravir for Oral Treatment of COVID-19 in Nonhospitalized Patients. N. Engl. J. Med..

[66] Corritori S., Savchuk N., Pauza C.D. (2022). Risk/Benefit Profiles of Currently Approved Oral Antivirals for Treatment of COVID-19: Similarities and Differences. Covid.

[67] Alhumaid S., Al Mutair A., Al Alawi Z., Alhmeed N., Zaidi A.R.Z., Tobaiqy M. (2020). Efficacy and Safety of Lopinavir/Ritonavir for Treatment of COVID-19: A Systematic Review and Meta-Analysis. Trop. Med. Infect. Dis..

[68] Ma L., Xie Y., Zhu M., Yi D., Zhao J., Guo S., Zhang Y., Wang J., Li Q., Wang Y. (2022). Identification of Darunavir Derivatives for Inhibition of SARS-CoV-2 3CL^pro^. Int. J. Mol. Sci..

[69] Ahmad B., Batool M., Ain Q.U., Kim M.S., Choi S. (2021). Exploring the Binding Mechanism of PF-07321332 SARS-CoV-2 Protease Inhibitor through Molecular Dynamics and Binding Free Energy Simulations. Int. J. Mol. Sci..

[70] Zhou Y., Wang H., Yang L., Wang Q. (2022). Progress on COVID-19 Chemotherapeutics Discovery and Novel Technology. Molecules.

[71] Mudatsir M., Yufika A., Nainu F., Frediansyah A., Megawati D., Pranata A., Mahdani W., Ichsan I., Dhama K., Harapan H. (2020). Antiviral Activity of Ivermectin Against SARS-CoV-2: An Old-Fashioned Dog with a New Trick—A Literature Review. Sci. Pharm..

[72] Samaha A.A., Mouawia H., Fawaz M., Hassan H., Salami A., Bazzal A.A., Saab H.B., Al-Wakeel M., Alsaabi A., Chouman M. (2021). Retraction: Samaha et al. Effects of a Single Dose of Ivermectin on Viral and Clinical Outcomes in Asymptomatic SARS-CoV-2 Infected Subjects: A Pilot Clinical Trial in Lebanon. Viruses 2021, 13, 989. Viruses.

[73] Kumar S., Singh B., Kumari P., Kumar P.V., Agnihotri G., Khan S., Kant Beuria T., Syed G.H., Dixit A. (2021). Identification of multipotent drugs for COVID-19 therapeutics with the evaluation of their SARS-CoV2 inhibitory activity. Comput. Struct. Biotechnol. J..

[74] Castillejos-López M., Torres-Espíndola L.M., Huerta-Cruz J.C., Flores-Soto E., Romero-Martinez B.S., Velázquez-Cruz R., Higuera-Iglesias A., Camarena Á., Torres-Soria A.K., Salinas-Lara C. (2022). K.; Salinas-Lara, C.; et al. Ivermectin: A Controversial Focal Point during the COVID-19 Pandemic. Life.

[75] Chan-Tack K., Sampson M., Earp J., Arya V., Yao L., Alexander J., Hausman E., Belew Y., Birnkrant D., Struble K. (2022). Considerations and Challenges in the Remdesivir COVID-19 Pediatric Development Program. J. Clin. Pharmacol..

[76] Plante K.S., Dwivedi V., Plante J.A., Fernandez D., Mirchandani D., Bopp N., Aguilar P.V., Park J.-G., Tamayo P.P., Delgado J. (2021). Antiviral activity of oleandrin and a defined extract of Nerium oleander against SARS-CoV-*Biomed*. Pharmacother..

[77] Kino T., Burd I., Segars J.H. (2021). Dexamethasone for Severe COVID-19: How Does It Work at Cellular and Molecular Levels?. Int. J. Mol. Sci..

[78] Romanou V., Koukaki E., Chantziara V., Stamou P., Kote A., Vasileiadis I., Koutsoukou A., Rovina N. (2021). Dexamethasone in the Treatment of COVID-19: Primus Inter Pares?. J. Pers. Med..

[79] Calzetta L., Aiello M., Frizzelli A., Rogliani P., Chetta A. (2021). Dexamethasone in Patients Hospitalized with COVID-19: Whether, When and to Whom. J. Clin. Med..

[80] Maláska J., Stašek J., Duška F., Balík M., Máca J., Hruda J., Vymazal T., Klementová O., Zatloukal J., Gabrhelík T. (2022). Effect of dexamethasone in patients with ARDS and COVID-19 (REMED trial)—Study protocol for a prospective, multi-centre, open-label, parallel-group, randomized controlled trial. Trials.

[81] Mahdi M., Hermán L., Réthelyi J.M., Bálint B.L. (2022). Potential Role of the Antidepressants Fluoxetine and Fluvoxamine in the Treatment of COVID-19. Int. J. Mol. Sci..

[82] Sukhatme V.P., Reiersen A.M., Vayttaden S.J., Sukhatme V.V. (2021). Fluvoxamine: A Review of Its Mechanism of Action and Its Role in COVID-19. Front. Pharmacol..

[83] Aldén M., Olofsson Falla F., Yang D., Barghouth M., Luan C., Rasmussen M., De Marinis Y. (2022). Intracellular Reverse Transcription of Pfizer BioNTech COVID-19 mRNA Vaccine BNT162b2 In Vitro in Human Liver Cell Line. Curr. Issues Mol. Biol..

[84] Balasubramaniyam A., Ryan E., Brown D., Hamza T., Harrison W., Gan M., Sankhala R.S., Chen W.-H., Martinez E.J., Jensen J.L. (2023). Unglycosylated Soluble SARS-CoV-2 Receptor Binding Domain (RBD) Produced in *E. coli* Combined with the Army Liposomal Formulation Containing QS21 (ALFQ) Elicits Neutralizing Antibodies against Mismatched Variants. Vaccines.

[85] Ghimire D., Han Y., Lu M. (2022). Structural Plasticity and Immune Evasion of SARS-CoV-2 Spike Variants. Viruses.

[86] McGill A.R., Kahlil R., Dutta R., Green R., Howell M., Mohapatra S., Mohapatra S.S. (2021). SARS-CoV-2 Immuno-Pathogenesis and Potential for Diverse Vaccines and Therapies: Opportunities and Challenges. Infect. Dis. Rep..

[87] Peng M.-Y., Liu W.-C., Zheng J.-Q., Lu C.-L., Hou Y.-C., Zheng C.-M., Song J.-Y., Lu K.-C., Chao Y.-C. (2021). Immunological Aspects of SARS-CoV-2 Infection and the Putative Beneficial Role of Vitamin-D. Int. J. Mol. Sci..

[88] Coban M.A., Morrison J., Maharjan S., Hernandez Medina D., Li W., Zhang Y.S., Freeman W.D., Radisky E.S., Le Roch K.G., Weisend C.M. (2021). Attacking COVID-19 Progression Using Multi-Drug Therapy for Synergetic Target Engagement. Biomolecules.

[89] Holms R.D. (2022). Long COVID (PASC) Is Maintained by a Self-Sustaining Pro-Inflammatory TLR4/RAGE-Loop of S100A8/A9 > TLR4/RAGE Signalling, Inducing Chronic Expression of IL-1b, IL-6 and TNFa: Anti-Inflammatory Ezrin Peptides as Potential Therapy. Immuno.

[90] Saber-Ayad M., Hammoudeh S., Abu-Gharbieh E., Hamoudi R., Tarazi H., Al-Tel T., Hamid Q. (2021). Current Status of Baricitinib as a Repurposed Therapy for COVID-19. Pharmaceuticals.

[91] A García-García J., Pérez-Quintana M., Ramos-Giráldez C., Cebrián-González I., Martín-Ponce M.L., del Valle-Villagrán J., A Navarro-Puerto M., Sánchez-Villegas J., Gómez-Herreros R., Manoja-Bustos I. (2021). Anakinra versus Baricitinib: Different Strategies for Patients Hospitalized with COVID-19. J. Clin. Med..

[92] Kojima Y., Nakakubo S., Takei N., Kamada K., Yamashita Y., Nakamura J., Matsumoto M., Horii H., Sato K., Shima H. (2022). Comparative Efficacy of Tocilizumab and Baricitinib Administration in COVID-19 Treatment: A Retrospective Cohort Study. Medicina.

[93] Richardson P.J., Robinson B.W.S., Smith D.P., Stebbing J. (2022). The AI-Assisted Identification and Clinical Efficacy of Baricitinib in the Treatment of COVID-19. Vaccines.

[94] Atluri K., Aimlin I., Arora S. (2022). Current Effective Therapeutics in Management of COVID-19. J. Clin. Med..

[95] Vannucchi A.M., Mortara A., D’Alessio A., Morelli M., Tedeschi A., Festuccia M.B., Monforte A.D., Capochiani E., Selleri C., Simonetti F. (2021). JAK Inhibition with Ruxolitinib in Patients with COVID-19 and Severe Pneumonia: Multicenter Clinical Experience from a Compassionate Use Program in Italy. J. Clin. Med..

[96] Gatti M., Turrini E., Raschi E., Sestili P., Fimognari C. (2021). Janus Kinase Inhibitors and Coronavirus Disease (COVID)-19: Rationale, Clinical Evidence and Safety Issues. Pharmaceuticals.

[97] Atzeni F., Masala I.F., Rodríguez-Carrio J., Ríos-Garcés R., Gerratana E., La Corte L., Giallanza M., Nucera V., Riva A., Espinosa G. (2021). The Rheumatology Drugs for COVID-19 Management: Which and When?. J. Clin. Med..

[98] Ong S.W.X., Ren D., Lee P.H., Sutjipto S., Dugan C., Khoo B.Y., Tay J.X., Vasoo S., Young B.E., Lye D.C. (2022). Real-World Use of Sotrovimab for Pre-Emptive Treatment in High-Risk Hospitalized COVID-19 Patients: An Observational Cross-Sectional Study. Antibiotics.

[99] Biscarini S., Villa S., Genovese C., Tomasello M., Tonizzo A., Fava M., Iannotti N., Bolis M., Mariani B., Valzano A.G. (2022). Safety Profile and Outcomes of Early COVID-19 Treatments in Immunocompromised Patients: A Single-Centre Cohort Study. Biomedicines.

[100] Quiros-Roldan E., Amadasi S., Zanella I., Degli Antoni M., Storti S., Tiecco G., Castelli F. (2021). Monoclonal Antibodies against SARS-CoV-2: Current Scenario and Future Perspectives. Pharmaceuticals.

[101] Cicchitto G., Cardillo L., de Martinis C., Sabatini P., Marchitiello R., Abate G., Rovetti A., Cavallera A., Apuzzo C., Ferrigno F. (2022). Effects of Casirivimab/Imdevimab Monoclonal Antibody Treatment among Vaccinated Patients Infected by SARS-CoV-2 Delta Variant. Viruses.

[102] Hall J., Salama M. (2022). Survival Benefit of Tocilizumab in COVID-19 May Be Greater in Patients with Higher Measured Interleukin 6 Levels. COVID.

[103] Conti V., Corbi G., Sellitto C., Sabbatino F., Maci C., Bertini N., De Bellis E., Iuliano A., Davinelli S., Pagliano P. (2021). Effect of Tocilizumab in Reducing the Mortality Rate in COVID-19 Patients: A Systematic Review with Meta-Analysis. J. Pers. Med..

[104] Vena A., Cenderello G., Balletto E., Mezzogori L., Santagostino Barbone A., Berruti M., Ball L., Battaglini D., Bonsignore A., Dentone C. (2021). Early Administration of Bamlanivimab in Combination with Etesevimab Increases the Benefits of COVID-19 Treatment: Real-World Experience from the Liguria Region. J. Clin. Med..

[105] Focosi D., Tuccori M. (2022). Prescription of Anti-Spike Monoclonal Antibodies in COVID-19 Patients with Resistant SARS-CoV-2 Variants in Italy. Pathogens.

[106] Plichta J., Kuna P., Panek M. (2022). Monoclonal Antibodies as Potential COVID-19 Therapeutic Agents. COVID.

[107] Drouin A.C., Theberge M.W., Liu S.Y., Smither A.R., Flaherty S.M., Zeller M., Geba G.P., Reynaud P., Rothwell W.B., Luk A.P. (2021). Successful Clearance of 300 Day SARS-CoV-2 Infection in a Subject with B-Cell Depletion Associated Prolonged (B-DEAP) COVID by REGEN-COV Anti-Spike Monoclonal Antibody Cocktail. Viruses.

[108] Focosi D., Casadevall A. (2022). A Critical Analysis of the Use of Cilgavimab plus Tixagevimab Monoclonal Antibody Cocktail (Evusheld™) for COVID-19 Prophylaxis and Treatment. Viruses.

[109] Awadasseid A., Yin Q., Wu Y., Zhang W. (2021). Potential protective role of the anti-PD-1 blockade against SARS-CoV-2 infection. Biomed. Pharmacother..

[110] Félix L., Correia R., Sequeira R., Ribeiro C., Monteiro S., Antunes L., Silva J., Venâncio C., Valentim A. (2021). MS-222 and Propofol Sedation during and after the Simulated Transport of Nile tilapia (*Oreochromis niloticus*). Biology.

[111] Beghini D.G., Horita S.I., Henriques-Pons A. (2021). Mesenchymal Stem Cells in the Treatment of COVID-19, a Promising Future. Cells.

[112] Karakaş N., Üçüncüoğlu S., Uludağ D., Karaoğlan B.S., Shah K., Öztürk G. (2022). Mesenchymal Stem Cell-Based COVID-19 Therapy: Bioengineering Perspectives. Cells.

[113] Câmara D.A.D., Porcacchia A.S., Lizier N.F., De-Sá-Júnior P.L. (2021). A COVID-19 Overview and Potential Applications of Cell Therapy. Biologics.

[114] De Masi L., Argenio M.A., Giordano D., Facchiano A. (2022). Molecular Aspects of Spike–ACE2 Interaction. Encyclopedia.

[115] Yalcin H.C., Sukumaran V., Al-Ruweidi M.K.A.A., Shurbaji S. (2021). Do Changes in *ACE-2* Expression Affect SARS-CoV-2 Virulence and Related Complications: A Closer Look into Membrane-Bound and Soluble Forms. Int. J. Mol. Sci..

[116] Pang X.C., Zhang H.X., Zhang Z., Rinkiko S., Cui Y.M., Zhu Y.Z. (2021). The Two-Way Switch Role of ACE2 in the Treatment of Novel Coronavirus Pneumonia and Underlying Comorbidities. Molecules.

[117] Qu L., Chen C., Yin T., Fang Q., Hong Z., Zhou R., Tang H., Dong H. (2021). ACE2 and Innate Immunity in the Regulation of SARS-CoV-2-Induced Acute Lung Injury: A Review. Int. J. Mol. Sci..

[118] Triposkiadis F., Xanthopoulos A., Giamouzis G., Boudoulas K.D., Starling R.C., Skoularigis J., Boudoulas H., Iliodromitis E. (2021). ACE2, the Counter-Regulatory Renin–Angiotensin System Axis and COVID-19 Severity. J. Clin. Med..

[119] Tan M.I., Alfarafisa N.M., Septiani P., Barlian A., Firmansyah M., Faizal A., Melani L., Nugrahapraja H. (2022). Potential Cell-Based and Cell-Free Therapy for Patients with COVID-19. Cells.

[120] Rapin A., Noujaim P.-J., Taiar R., Carazo-Mendez S., Deslee G., Jolly D., Boyer F.C. (2022). Characteristics of COVID-19 Inpatients in Rehabilitation Units during the First Pandemic Wave: A Cohort Study from a Large Hospital in Champagne Region. Biology.

[121] Crum R.J., Capella-Monsonís H., Badylak S.F., Hussey G.S. (2022). Extracellular Vesicles for Regenerative Medicine Applications. Appl. Sci..

[122] Nalesso F., Garzotto F., Cattarin L., Gobbi L., Qassim L., Sgarabotto L., Tiberio I., Calò L.A. (2020). A Continuous Renal Replacement Therapy Protocol for Patients with Acute Kidney Injury in Intensive Care Unit with COVID-19. J. Clin. Med..

[123] Chávez-Valencia V., Orizaga-De-La-Cruz C., Lagunas-Rangel F.A. (2022). Acute Kidney Injury in COVID-19 Patients: Pathogenesis, Clinical Characteristics, Therapy, and Mortality. Diseases.

[124] Ruggiero V., Aquino R.P., Del Gaudio P., Campiglia P., Russo P. (2022). Post-COVID Syndrome: The Research Progress in the Treatment of Pulmonary *sequelae* after COVID-19 Infection. Pharmaceutics.

[125] Souri M., Chiani M., Farhangi A., Mehrabi M.R., Nourouzian D., Raahemifar K., Soltani M. (2022). Anti-COVID-19 Nanomaterials: Directions to Improve Prevention, Diagnosis, and Treatment. Nanomaterials.

[126] Ramezani Z., Dayer M.R., Noorizadeh S., Thompson M. (2021). Deactivation of SARS-CoV-2 via Shielding of Spike Glycoprotein Using Carbon Quantum Dots: Bioinformatic Perspective. COVID.

[127] He Q., Lu J., Liu N., Lu W., Li Y., Shang C., Li X., Hu L., Jiang G. (2022). Antiviral Properties of Silver Nanoparticles against SARS-CoV-2: Effects of Surface Coating and Particle Size. Nanomaterials.

[128] Merkl P., Long S., McInerney G.M., Sotiriou G.A. (2021). Antiviral Activity of Silver, Copper Oxide and Zinc Oxide Nanoparticle Coatings against SARS-CoV-2. Nanomaterials.

[129] Mosselhy D.A., Kareinen L., Kivistö I., Aaltonen K., Virtanen J., Ge Y., Sironen T. (2021). Copper-Silver Nanohybrids: SARS-CoV-2 Inhibitory Surfaces. Nanomaterials.

[130] Al-Hindawi A., AlDallal U., Waly Y.M., Hussain M.H., Shelig M., Saleh ElMitwalli O.S.M.M., Deen G.R., Henari F.Z. (2022). An Exploration of Nanoparticle-Based Diagnostic Approaches for Coronaviruses: SARS-CoV-2, SARS-CoV and MERS-CoV. Nanomaterials.

[131] Ardekani L.S., Thulstrup P.W. (2022). Gold Nanoparticle-Mediated Lateral Flow Assays for Detection of Host Antibodies and COVID-19 Proteins. Nanomaterials.

[132] Pranskuniene Z., Balciunaite R., Simaitiene Z., Bernatoniene J. (2022). Herbal Medicine Uses for Respiratory System Disorders and Possible Trends in New Herbal Medicinal Recipes during COVID-19 in Pasvalys District, Lithuania. Int. J. Environ. Res. Public Health.

[133] Mamedov T., Yuksel D., Ilgın M., Gürbüzaslan I., Gulec B., Mammadova G., Ozdarendeli A., Yetiskin H., Kaplan B., Islam Pavel S.T. (2021). Production and Characterization of Nucleocapsid and RBD Cocktail Antigens of SARS-CoV-2 in *Nicotiana benthamiana* Plant as a Vaccine Candidate against COVID-19. Vaccines.

[134] Ruocco V., Strasser R. (2022). Transient Expression of Glycosylated SARS-CoV-2 Antigens in *Nicotiana benthamiana*. Plants.

[135] Lim C.L., Raju C.S., Mahboob T., Kayesth S., Gupta K.K., Jain G.K., Dhobi M., Nawaz M., Wilairatana P., de Lourdes Pereira M. (2022). Precision and Advanced Nano-Phytopharmaceuticals for Therapeutic Applications. Nanomaterials.

